# Elevated IgM and abnormal free light chain ratio are increased in relatives from high-risk chronic lymphocytic leukemia pedigrees

**DOI:** 10.1038/s41408-019-0186-8

**Published:** 2019-02-26

**Authors:** Martha J. Glenn, Michael J. Madsen, Ethan Davis, Cassandra D. Garner, Karen Curtin, Brandt Jones, Justin A. Williams, Michael H. Tomasson, Nicola J. Camp

**Affiliations:** 1 University of Utah School of Medicine, Salt Lake City, UT 84112 USA; 2 Huntsman Cancer Institute, University of Utah, Salt Lake City, UT 84112 USA; 3 Carver College of Medicine, University ofIowa, Iowa City, IA 52242 USA

## Abstract

Abnormal serum immunoglobulin (Ig) free light chains (FLC) are established biomarkers of early disease in multiple B-cell lymphoid malignancies, including chronic lymphocytic leukemia (CLL). Heavy chains have also been shown to be biomarkers in plasma cell disorders. An unanswered question is whether these Ig biomarkers are heritable, i.e., influenced by germline factors. CLL is heritable but highly heterogeneous. Heritable biomarkers could elucidate steps of disease pathogenesis that are affected by germline factors, and may help partition heterogeneity and identify genetic pleiotropies across malignancies. Relatives in CLL pedigrees present an opportunity to identify heritable biomarkers. We compared FLCs and heavy chains between relatives in 23 high-risk CLL pedigrees and population controls. Elevated IgM (eIgM) and abnormal FLC (aFLC) ratio was significantly increased in relatives, suggesting that these Ig biomarkers are heritable and could offer risk stratification in pedigree relatives. Within high-risk CLL pedigrees, B-cell lymphoid malignancies were five times more prevalent in close relatives of individuals with eIgM, prostate cancer was three times more prevalent in relatives of individuals with aFLC, and monoclonal B-cell lymphocytosis increased surrounding individuals with normal Ig levels. These different clustering patterns suggest Ig biomarkers have the potential to partition genetic heterogeneity in CLL and provide insight into distinct heritable pleiotropies associated with CLL.

## Introduction

Chronic lymphocytic leukemia (CLL) is the most common leukemia affecting adults diagnosed in the United States (4.7/100,000 per year), with similar incidence in other western countries. There are very few known risk factors for CLL. These include family history, sex, race/ethnicity (risk is greatest in non-Hispanic White men^1^), and exposure to certain chemicals^2^. Of these, family history is the strongest risk factor, with large population-based studies consistently suggesting CLL as among the most familial cancers studied^3,4^ and suggesting a striking 5.7–7.8 increased risk in first-degree relatives^5–7^.

Biomarkers are biological traits associated with risk to a disease. Heritable biomarkers are those that share germline genetic factors with the disease and can shed light on the steps along disease pathogenesis affected by germline genetics. Heritable biomarkers have the potential to partition genetic heterogeneity (different routes to disease) and identify germline pleiotropies (same heritable biomarker, different disease endpoint). In complex diseases, heterogeneity is a key obstacle that challenges genetic discoveries, and hence the deconstruction of heterogeneity and definition of important pleiotropies will aid in the elucidatation of germline risk factors. Of clinical benefit, knowledge of early disease processes have been shown to be associated with better overall survival in multiple myeloma^8^. Future clinical benefit of heritable biomarkers in CLL could include risk stratification in relatives of diseased individuals toward prevention, early detection, and improved outcomes. A better understanding of heritable immune phenotypes also may have future potential to contribute to the growing knowledgebase concerning immunophenotypes and response to immunotherapies ^9^.

High-risk CLL pedigrees are families containing a significant excess of relatives with CLL. Focusing on “at-risk” relatives in CLL families is a powerful design to explore genetically controlled disease initiation. In particular, “at-risk” relatives can provide novel insight into heritable biomarkers. Monoclonal B-cell lymphocytosis (MBL), e.g., is the existence of small clones in peripheral blood (< 5 × 10^9^ clonal B-cells/L) and is a precursor to CLL^10^. High-risk CLL pedigrees have shown that MBL is a common occurrence (17%) in otherwise unaffected first-degree relatives^11^, compared with only 3–5% in the general population using comparable laboratory detection methods^12,13^. Thus, MBL is a heritable precursor sharing genetic susceptibility with CLL and indicates that inherited risk includes propensity for clonal development.

B-cells produce immunoglobulin (Ig) as part of the native and adaptive immune system. Aberrant B-cell activation and expansion may be reflected in abnormal polyclonal or monoclonal production of Ig heavy chains (e.g., IgA, IgG, and IgM) and/or light chains (κ and λ). This includes intact Igs comprising bound heavy light chains (HLC) and unbound free light chains (FLC) due to more abundant light-chain synthesis. Abnormal polyclonal (κ + λ) or monoclonal (κ/λ) FLCs in serum have been shown to be biomarkers of prognosis and survival in plasma cell disorders^14–18^ and other B-cell lymphoid malignancies, including CLL^19–24^. Prospective cohorts have identified elevated polyclonal and abnormal monoclonal FLCs as early biomarkers of CLL, detectable upto 9.8 years before diagnosis^25^. More recently, assays to quantitate serum Ig HLCs have been developed^26^. In plasma cell disorders, HLCs have also proven to be useful biomarkers of progression, prognosis, and survival, independent of FLC markers^18,27–32^. The role of HLC biomarkers has yet to be explored in other B-cell malignancies. Here we explore the hypothesis that inherited risk extends to early steps in CLL pathogenesis, defective immune response (polyclonal expansion), and the development of clonal populations. We investigate the potential for heritability of polyclonal and monoclonal FLCs and HLCs using “at-risk” relatives in CLL pedigrees.

## Materials and methods

### Case definition

CLL is characterized by infiltration of the bone marrow by a clonal population of mature, normal appearing but immunophenotypically abnormal B-cell lymphocytes. It is diagnosed by the presence of at least 5 × 10^9^ clonal B-cells/L in the peripheral blood, but the lymph nodes, spleen, and liver may also be involved. If diagnosed instead by clonal cells in the lymph nodes, it may be referred to as small lymphocytic lymphoma (SLL). These are considered the same malignancy and we use “CLL” hereafter as an abbreviation to refer to either CLL (International Classification of Disease for Oncology version 3 [ICD-O-3] histology code 9823/3) or SLL (ICD-O-3 9670/3). Relatives of CLL cases identified from the state-wide Utah Cancer Registry (UCR) and who were members in high-risk pedigrees (see below) were the focus of this study.

### High-risk pedigrees and controls

We previously ascertained high-risk CLL pedigrees using the Utah Population Database (UPDB)^11^. The UPDB record links a 5 million-person genealogy to the UCR, a state-wide registry since 1966, which has been part of the National Cancer Institute Surveillance, Epidemiology, and End Results Program since its inception. High-risk pedigrees are defined as those containing a statistical excess of CLL (*p* < 0.05), compared with sex-, birth cohort-, and birth place-matched rates from the full UPDB dataset. Biospecimens for living CLL cases and their relatives were previously collected in 23 high-risk pedigrees for genetic study. Serum samples were available for 117 relatives of CLL cases; mostly first degree (98%). At the time of sampling, some pedigree relatives were known to have other B-cell lymphoid malignancies, solid tumors, or screen-detected MBL^11^. Table 1 summarizes pedigree characteristics and Figure S1 illustrates the 23 pedigrees. Serum samples for 160 population controls with no personal history of hematological malignancies were also available. Some controls were known to have solid tumors at the time of sampling. All sampled relatives and controls provided informed consent and self-reported as White, non-Hispanic. All ascertainment was performed under University of Utah institutional review board approvals.

**Table 1:** Description of high-risk CLL pedigrees

Pedigree	Members	Generations	CLL	Sampled relatives	Heme malignancies	B-cell lymphoid malignancies	Solid tumors
6204	57	6	4	3	1	1	9
6205	71	7	7	3	0	0	6
6207	285	9	26	4	3	3	31
6208	62	6	5	4	1	1	4
6210	40	5	4	3	0	0	4
6211	189	7	15	10	1	0	33
6213	20	3	2	1	1	0	4
6215	36	5	4	6	0	0	0
6217	17	3	3	8	1	1	3
6218	42	6	4	2	1	0	5
6222	12	3	4	1	1	0	2
6223	47	5	3	4	1	1	3
6224	21	4	3	2	0	0	3
6225	32	4	4	4	0	0	8
6226	85	6	7	10	0	0	10
6228	130	7	12	7	2	2	18
6229	120	6	11	2	2	1	19
6232	25	4	2	8	3	3	1
6233	247	7	24	14	8	6	26
6234	218	6	19	6	6	4	29
6235	46	6	4	6	1	1	4
6236	34	5	2	8	1	1	2
6239	5	2	2	1	0	0	0
**TOTAL**	**1841**		**171**	**117**	**34**	**25**	**224**

Pedigree structures were defined to include the following: all known CLL cases from the UPDB that determined the high-risk status, all siblings of the CLL cases, all sampled relatives used in this study and their siblings, and all connecting relatives to the common ancestral founder/s. Column headings: Members refer to the total number of individuals in each pedigree structure; Generations is the number of generations from the pedigree founder/s; CLL indicates the number of individuals diagnosed with CLL or SLL in the pedigree; Sampled relatives indicate the number of relatives with serum used in this study; Heme malignancies represent a count of bloodline pedigree members (i.e., not including marry-ins) in each pedigree with a UCR-confirmed hematological malignancy (not including CLL/SLL); B-cell lymphoid malignancies indicate the number of bloodline pedigree members with a UCR-confirmed B-cell lymphoid malignancy (not including CLL/SLL); and Solid tumors indicate the count of UCR-confirmed solid tumors in bloodline pedigree members. Note: individuals with multiple cancers will be counted more than once

### Ig measurements

Serum was extracted from whole blood samples collected in red-top Vacutainer tubes. Blood samples were allowed to clot at room temperature for ~20 min after which the clot was removed by centrifugation at 1000–2000  *g-force* for 10 min. The resulting serum was transferred to polypropylene tubes and stored at − 20 °C. Stored serum was thawed on ice and concentrations of FLCs (κ and λ) and isotype-specific HLCs (IgAκ, IgAλ, IgGκ, IgGλ, IgMκ, and IgMλ) were measured at The Binding Site Ltd (Birmingham, UK) with Freelite® and Hevylite® assays, respectively. The Freelite® is a well-established assay that gained wide use in the diagnosis, treatment assessment, and disease monitoring of plasma cell disorders. The newer Hevylite® assay allows for quantification of IgM, IgA, and IgG bound to specific light chains using serum markers for isotype-specific Ig HLC junction epitopes ^26^.

Variables indicative of polyclonal and monoclonal B-cell expansion were considered. Quantitative variables for polyclonality were the summation of κ and λ, either unbound (free) or bound to specific heavy chains: FLC(κ + λ), IgM(κ + λ), IgA(κ + λ), and IgG(κ + λ). Dichotomous variables indicative of clonal development were as follows: abnormal FLC (aFLC) ratio (κ/λ) and abnormal HLC ratios (IgM(κ/λ), IgA(κ/λ), and IgG(κ/λ)). The use of the FLC ratio is clinically established and an aFLC ratio was defined as one beyond its diagnostic range (0.26–1.65)^33^. Abnormal HLC ratios were defined as those beyond the 95% reference range in controls: 0.84–1.91 for IgA(κ/λ); 1.02–2.87 for IgG(κ/λ); and 1.00–2.90 for IgM(κ/λ), which are similar to other published ranges for HLC ratios ^26,34^.

### Statistical analyses

For quality control, individuals with Ig values >5 SDs beyond the mean for their group were excluded as outliers. Propensity-score adjustment with optimal match selection on sex and age was used to control imbalances and nonlinearity in sex and age distributions between the relatives and the controls^35^. Associations between each Ig variable and “at-risk” relative status were performed using propensity-score-adjusted linear (for continuous polyclonal variables) and logistic (for dichotomous monoclonal variables) regression models. Firth’s bias reduction method was employed for estimation of logistic regression parameters to address low observed counts^36^. To explore the independence of multiple Ig variables identified from univariable analyses, multivariable models with correction for age and sex were constructed. Although increases in “at-risk” relatives are of particular interest (consistent with our hypothesis regarding heritability), all tests were two-sided. A nominal type 1 error threshold (*a* = 0.05) for statistical significance was used throughout.

For Ig phenotypes found to be enriched in relatives, we further performed pedigree-only analyses (not including the control data). We categorized pedigrees as harboring aberrant Ig phenotypes or not. Based on these pedigree categories, we explored cancers in the relatives surrounding individuals with abnormal Ig phenotypes and tested differences between the pedigree categories. Such comparisons can identify cancer spectra that share germline susceptibility with biomarkers of CLL and differences may provide insight into CLL genetic heterogeneity. These pedigree comparisons were possible due to the unique UPDB resource, which provides detailed cancer family history for individuals (both sampled and unsampled) within the high-risk pedigrees via record-linkage to the UCR; a state-wide surveillance of cancer began in Utah in 1966. Cancers observed in pedigree individuals with aberrant Ig phenotypes and their first- to third-degree relatives were counted (individuals that married-in to the pedigree were excluded). These were compared with cancer counts in and surrounding individuals with normal Ig phenotypes in pedigrees without any aberrant phenotypes. First- to third-degree relatives (i.e., out to cousins) were considered to balance contributions across pedigrees, as some pedigrees are much larger than others (Figure S1). Differences were illustrated using odds ratios (ORs) with 95% confidence intervals (CIs), using Fisher’s method to address small counts.

## Results

One control individual was removed as an outlier, resulting in a set of 117 at-risk relatives and 159 population controls for analysis. Summaries for the demographics for these two groups are shown in Table 2 and their Ig values are shown in Table 3.

**Table 2:** Demographics for “at-risk” relatives and population controls

Group	*n*	Age in years, median (IQR)	% Males
High-risk pedigree relatives^a^	117	58 (50–75)	47.0
Cancer-free	99	56 (48–69)	41.4
Solid tumors	14	76 (65–82)	78.6
B-cell lymphoid malignancies^b^	4	75 (71–77)	75.0
Controls	159	67 (52–82)	54.1
Cancer-free	152	68 (61–76)	54.6
Solid tumors	7	64 (60–69)	42.9

Age indicates age at sampling. IQR indicates the interquartile range (25^th^–75th percentile values). ^a^Status at time of sampling. ^b^B-cell lymphoid malignancies do not include CLL

**Table 3:** Immunoglobulin phenotypes by group

	Controls^a^		High-risk pedigree relatives^a^			
	All	Cancer-free	All	Cancer-free	Solid tumor	B-cell lymphoid malignancy^b^
*n*	159	153	117	99	14	4
κ + λ (mg/dL), median (95% range)						
FLC	3.04 (1.55–6.30)	3.00 (1.54–6.33)	2.62 (1.61–5.14)	2.60 (1.59–4.75)	2.91	3.94
IgA	210 (77–497)	206 (77–497)	207 (60–428)	209 (62–391)	184	100
IgG	1116 (682–1586)	1116 (672–1592)	1108 (613–1526)	1113 (638–1531)	1011	856
IgM	***68 (22***–***220)***	68 (22–220)	***92 (33***–***286)***	93 (31–301)	68	108
κ/λ ratio, % abnormal^c^						
FLC	***2 (1.3%)***	2 (1.3%)	***8 (6.8%)***	5 (5.1%)	2	1
IgA	8 (5.0%)	7 (4.6%)	7 (6.0%)	5 (5.1%)	2	0
IgG	8 (5.0%)	8 (5.3%)	12 (10.3%)	9 (9.1%)	2	1
IgM	8 (5.0%)	7 (4.6%)	3 (2.6%)	3 (3.4%)	0	0

Controls were selected to not have any hematological maliganacy. Sampled relatives in high-risk CLL pedigrees could not have CLL or SLL. ^a^Status at time of sampling. ^b^B-cell lymphoid malignancies do not include CLL; 95% range indicates the 2.5th–97.5th percentile range. ^c^FLC ratio is used clinically and abnormality is based on its established diagnostic range (0.26–1.65), otherwise abnormality is based on the HLC ratio 95% reference ranges from the controls as follows: 0.84–1.91 for IgA(κ/λ); 1.02–2.87 for IgG(κ/λ); and 1.00–2.90 for IgM(κ/λ). Table entries that are bold and italic indicate those that are statistically different between at-risk relatives and controls

### Association of “at-risk” relatives and Ig variables

Polyclonal variables were analyzed as quantitative traits. Results are shown in Table 4. A statistically significant difference in IgM levels was found between high-risk pedigree relatives and controls (*p* = 0.033, after adjusting for age and sex), indicating a 16.0 mg/dL increase in relatives (95% CI 1.4–30.5). Defining elevated IgM (eIgM) to be a value beyond the 95th percentile from the control reference range (IgM(κ + λ) ≥ 19 mg/dL), 11.1% of the “at-risk” relatives had eIgM (13/117). When individuals with known MBL, hematological malignancies, or solid tumors at the time of sampling were removed, results were similar and remained significant (data not shown). No statistically significant differences were found for polyclonal variables FLC(κ + λ), IgA(κ + λ), or IgG(κ + λ).

**Table 4:** Propensity-score adjusted regression analysis results

Polyclonal immunoglobulin measures		
**Measure**	**Difference in serum levels [mg/dL] in relatives (95% CI)**	***p*** **-Value**
FLC(κ + λ)	− 0.2 (− 0.6,0.2)	0.33
IgA(κ + λ)	− 15.8 (− 41.6,10)	0.23
IgG(κ+λ)	− 9.8 (− 69.1,49.5)	0.75
IgM(κ+λ)	***16.0 (1.3,30.6)***	***0.033***
Monoclonal immunoglobulin measures^a^		
**Measure**	**Fold difference for monoclonality in relatives (95% CI)**	***p*** **-Value**
FLC (κ/λ)	***6.38 (1.65,34.97)***	***0.0063***
IgA(κ/λ)	0.95 (0.3,2.76)	0.93
IgG(κ/λ)	1.44 (0.48,4.24)	0.51
IgM(κ/λ)	0.49 (0.12,1.52)	0.23

Propensity-score adjustment with matching on sex and age was used to control imbalances and nonlinearity in sex and age distributions between relatives and controls; *p*-values are from the Wald’s test. Polyclonal Ig variables are quantitative traits and are tested within a linear regression framework. Average differences in relatives compared with controls and 95% confidence intervals (CIs) are reported. Monoclonal Ig variables are dichotomous and tested within a logistic regression framework. Fold differences for monoclonality (abnormal κ/λ ratio, free, or bound) and 95% CI are reported from the logistic model. ^a^Abnormality for FLC ratio (κ/λ) is based on its established diagnostic range (0.26–1.65). Abnormal HLC ratios are based on the 95% reference range from controls as follows: 0.84–1.91 for IgA(κ/λ); 1.02–2.87 for IgG(κ/λ); and 1.00–2.90 for IgM(κ/λ). Table entries that are bold and italic indicate those that are statistically different between at-risk relatives and controls

**Fig. 1:**
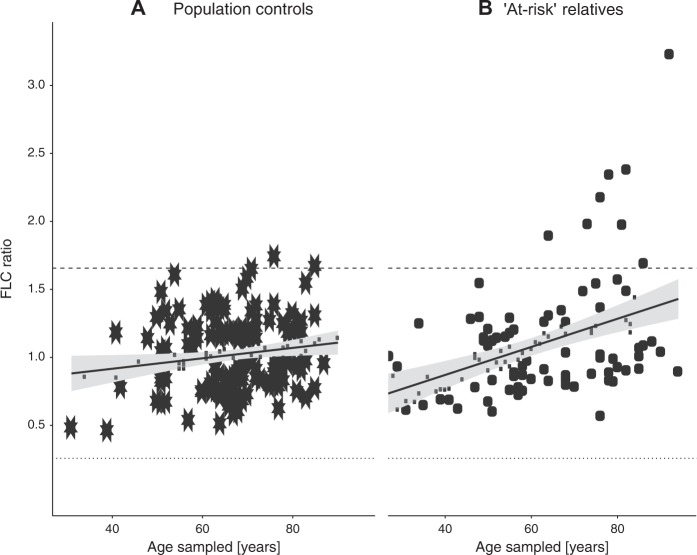
Free light chain (FLC) ratio by age at time of sample collection. **a** Population controls. **b** “At-risk” relatives in high-risk CLL pedigrees. Dashed/dotted lines indicate the upper and lower limit of the diagnostic range for FLC ratio (κ/λ, 0.26–1.65). Solid lines indicate the trend line fit by least squares regression

Monoclonality was determined by abnormal κ/λ ratios based on the established diagnostic range for the FLC ratio or control 95% reference ranges (for IgA, IgG, and IgM). Results are shown in Table 4. No statistically significant differences were found for monoclonality of any of the HLCs. aFLC ratios were significantly increased in the pedigree relatives compared with controls (OR = 6.38, 95% CI 1.65–34.97, *p* = 0.0063). The result remained similar and significant when individuals with known MBL or cancers were removed. Figure 1 illustrates FLC(κ/λ) ratio by age at sampling, showing a steeper trajectory in the “at-risk” relatives than in the controls. Increasing FLC ratio with age is highly significant for relatives (increase of 0.10 per 10 years, *p* < 0.001), a 2.7-fold higher rate of increase than in the population controls (0.038 per 10 years, *p* = 0.029).

To establish whether IgM level and aFLC ratio were independent biomarkers, we performed a multivariable analysis with adjustment for age and sex. Both eIgM and aFLC ratio were independent predictors and remained significant in the full model: eIgM OR = 1.61 (95% CI 1.04–2.58, *p* = 0.034) and aFLC ratio OR = 5.56 (95% CI 2.35–50.80, *p* = 0.0011).

### Comparison of cancer configurations within pedigrees

In pedigree-only analyses, we identified the pedigrees that harbored aberrant phenotypes and those that did not. Five pedigrees contained eight members with aFLC ratios. These 8 individuals together had 93 unique first- to third-degree relatives. Eight pedigrees contained 13 members with eIgM levels, who together had 132 first- to third-degree relatives. Cancers in these individuals/relatives were compared with 46 individuals with normal Ig phenotype and their 257 unique first- to third-degree relatives residing in 13 “normal” pedigrees (those not containing any members with abnormal Ig phenotypes). Table 5 summarizes cancer configuration comparisons between pedigrees. B-cell lymphoid malignancies (not including CLL or SLL) were 5 times more likely to be observed in the 145 relatives and individuals with eIgM compared with the 303 relatives and “normal” individuals (OR = 5.05, 95% CI 1.13–30.73; 4.8% vs. 1.0%). B-cell lymphoid malignancies were also more likely to be observed in and surrounding individuals with aFLC ratio than in the normal pedigrees (4.0% vs. 1.0%; OR = 4.15 95% CI 0.69–28.83), although not statistically significant. Surprisingly, even though all pedigrees were high risk for CLL by design, there was a greater proportion of relatives with CLL in “normal” pedigrees (14.8% vs. 11.4% and 7.6% surrounding individuals with eIgM and aFLC ratio, respectively). In addition, MBL was more likely to be observed in the first- to third-degree relatives and individuals with normal Ig phenotypes (4.6% compared with 3.0% and 0.7% in the individuals and relatives with aFLCr or eIgM, respectively). In terms of solid tumors, prostate cancer was over three times more likely to be observed in the male individuals with aFLC ratios and their male relatives (OR = 3.19, 95% CI 1.21–8.40; 21.8% vs. 8.0% in and around “normals”). In contrast, prostate cancer was not increased in relatives and individuals with eIgM (OR = 0.72; 5.9% vs. 8.0%). The observations of increased hematological malignancies (eIgM and aFLC ratio) and prostate cancer (aFLC ratio only) remained very similar when the personal cancer history of the individuals themselves were removed from the comparison.

**Table 5:** Cancers observed in individuals with eIgM and aFLC ratio and their relatives compared with those in individuals with normal Ig phenotypes and their relatives

	Normal^a^	aFLC ratio		eIgM	
		Count (%)	OR, 95% CI	Count (%)	OR, 95% CI
Pedigrees	13	5		8	
Index individuals	46	8		13	
**Total 1st-3rd degree relatives**	**257**	**92**		**132**	
**CLL**	38 (14.8%)	7 (7.6%)	0.48, 0.17–1.13	15 (11.4%)	0.74, 0.36–1.44
**Total index** **+** **relatives (males:females)**	**303 (150:153)**	**101 (55:46)**		**145 (85:60)**	
**MBL**	***14 (4.6%)***	3 (3.0%)	0.64, 0.12–2.36	***1 (0.7%)***	***0.14, 0.00–0.96***
**Hematological malignancies (not incl CLL)**					
Hematological malignancies	5 (1.7%)	5 (5.0%)	3.13, 0.70–13.89	8 (5.5%)	3.47, 0.98–13.74
B-cell lymphoid malignancies	***3 (1.0%)***	4 (4.0%)	4.15, 0.69–28.83	***7 (4.8%)***	***5.05, 1.13–30.73***
**Solid tumors, number (%)**					
Prostate	***12 (8.0%)***	***12 (21.8%)***	***3.19, 1.21–8.40***	5 (5.9%)	0.72, 0.19–2.29
Melanoma	7 (2.3%)	3 (3.0%)	1.31, 0.21–5.86	6 (4.1%)	1.82, 0.50–6.47
Colorectal	8 (2.6%)	1 (1.0%)	0.37, 0.01–2.84	1 (0.7%)	0.26, 0.01–1.94
Breast	7 (4.6%)	3 (6.7%)	1.49, 0.24–6.87	2 (3.3%)	0.72, 0.07–3.93
Urinary tract	1 (0.3%)	3 (3.0%)	9.28, 0.74–490.45	4 (2.8%)	8.52, 0.83–422.31
Gynaelogical	3 (2.0%)	2 (4.4%)	2.31, 0.19–20.88	1 (1.7%)	0.85, 0.02–10.81
Lung	2 (0.7%)	0 (0.0%)	nd	1 (0.7%)	1.05, 0.02–20.23
Pancreas	1 (0.3%)	0 (0.0%)	nd	2 (1.4%)	4.21, 0.22–249.60

^a^A “normal” pedigree indicates one that does not contain any members with abnormal Ig phenotypes in this study. Index individuals indicate those with eIgM, aFLC ratio, or those with normal Ig phenotypes (in “normal” pedigrees). *95% CI* 95% confidence interval, *OR* odds ratio, *nd* not determined (counts too low to assess). All ORs and CIs are from Fisher’s exact method. Percent CLL (in first- to third-degree relatives). Percent MBL and percent other cancers (in the sum of index individuals and first- to third-degree relatives). Supplementary Figure S1 illustrates the pedigree drawings and shows all the UCR-confirmed cancer diagnoses from which these summaries are made. All statistics for prostate, breast, and gynelogical cancer are sex specific

## Discussion

Family history is the strongest known risk factor for CLL^5–7^. The high-risk pedigree design is therefore an effective design to explore heritable biomarkers. Previously, a high-risk CLL pedigree design showed that MBL was a heritable precursor, significantly increased in pedigree relatives^11^, suggesting that a component of inherited risk may be to clonal development. Here we used a high-risk CLL pedigree design to investigate polyclonal and monoclonal Ig phenotypes as heritable biomarkers for CLL. We found that aFLC ratios and IgM levels were significantly increased in “at-risk” pedigree relatives compared with controls, and that these were independent factors.

A role for aFLC ratio as a precursor biomarker for CLL was first suggested in a prospective study, with monoclonality observed in 38% of individuals subsequently diagnosed with CLL, and detected upto 9.8 years, before diagnosis^25^. Our observation that aFLC ratio is more prevalent in “at-risk” relatives in pedigrees compared with controls (6.8% vs. 1.3%, OR = 6.38, *p* = 0.0063) is consistent with aFLC ratio as a heritable CLL precursor biomarker. Abnormal FLC ratio is used clinically to indicate clonal plasma cells in plasma cell disorders. There is also evidence that aFLC ratio is associated with survival and time to first treatment in CLL^19–23^, suggesting it as an important biomarker for clonal B-cell development. In the context of a precursor, a biomarker for active clonal development has the potential to capture important information on progression toward overt disease. Further investigation of aFLC ratio as an effective risk stratification tool for early disease, particularly in at-risk relatives, may be warranted.

HLCs have not previously been studied as a precursor or prognostic indicators for CLL. In this study, we found that polyclonal IgM was elevated in pedigree relatives compared with controls, and that this association was independent from a monoclonal FLC ratio. Serum IgM has several important roles, including the clearance of pathogens and apoptotic cells, and regulation of immune response^37^. Failure to remove dying cells is essential to prevent autoimmunity and inflammation^38^, and a role for natural IgM has been described in various autoimmune and inflammatory diseases^39^, and may contribute to the co-clustering that has been reported for B-cell malignancies and autoimmune disorders^40–42^. In Waldenstrom’s macroglobulinemia, a B-cell malignancy with similarities to CLL, evidence for polyclonal IgM levels was reported in unaffected relatives in high-risk families, some of which subsequently progressed to monoclonal IgM^43^. Interestingly, the most significant common risk allele for CLL from genome-wide association studies, rs735665-A at 11q24.1 (OR = 1.63, 95% CI 1.53–1.72)^44^, was recently associated with an increase in natural IgM levels^45^. Furthermore, secreted IgM has been shown to accelerate CLL tumor progression in mouse models^46^. It is notable, however, that although CLL tumors are predominantly IgM producing, it is generally membrane bound and not secreted into the serum. The significance of polyclonal IgM as a heritable precursor biomarker for CLL requires further investigation. Attention to IgM-secreting CLL patients regarding progression may also be warranted.

Our evidence for two new heritable biomarkers for CLL, elevated polyclonal IgM and monoclonal FLC ratio, suggests that genetic susceptibility may include defective immune response (polyclonal expansion) and development of clonal populations. Of further interest is whether these two biomarkers represent the same underlying genetic risk observed along the continuum of CLL development or different avenues of genetic risk (genetic heterogeneity). To begin to address this, we explored the individual and family cancer history of individuals with these two abnormal Ig phenotypes. We found that B-cell malignancies were significantly increased surrounding individuals with eIgM, compared with other high-risk pedigrees where eIgM was not apparent. This observation is consistent with overlapping genetic risk factor/s across hematological malignancies. Also consistent with this conjecture, was that pedigrees without eIgM (or aFLC) were more homogeneous for CLL (harboring both a higher proportion of CLL and MBL, and a lower proportion of other B-cell lymphoid malignancies). A unique feature to the family cancer history in and surrounding individuals with aFLC ratio was a significantly increased prevalence of prostate cancer. This was in striking contrast to the reduction of prostate cancer observed in and surrounding individuals with eIgM. Co-clustering of CLL and prostate cancer in families has been observed repeatedly^47–49^, and prostate cancers have been observed as increased secondary cancers in individuals with CLL^50–52^. Here we find that relatives with aFLC ratio partition our high-risk pedigrees into those exhibiting particularly strong co-clustering with prostate cancer and not, and suggests that it may represent a biomarker that parses shared genetic etiology between CLL and prostate cancer. Based on these observations, we hypothesize that eIgM and aFLC ratios are heritable biomarkers that represent separate genetic susceptibilities for CLL and as such may also be useful as a strategy to reduce heterogeneity in genetic mapping studies.

Our high-risk pedigree study has unique strengths, particularly the rich cancer family history available from the UPDB/UCR and the ability to study large, multi-generational families. It is limited in the fact that it is a single study, which is largely based on a White, non-Hispanic population. All findings will require replication elsewhere. Nonetheless, our novel findings complement other observations in the literature and the hypotheses generated provide new and exciting avenues for the field to consider.

In conclusion, we propose eIgM and aFLC ratios as two new heritable biomarkers for CLL representing separate genetic susceptibilites. Together with MBL, these biomarkers suggest that genetic risk involves susceptibility to polyclonal expansion, clonal development, and clonal expansion. These biomarkers could be useful for developing risk stratification strategies for relatives of CLL cases to identify early disease. In addition, these biomarkers in the relatives in pedigrees may offer a strategy to reduce genetic heterogeneity providing new opportunities to increase power in pedigree-based gene mapping studies. Continued longitudinal studies of high-risk pedigrees has the potential to continue to uncover important steps in the pathogenesis of CLL and other lymphoproliferative disorders.

## Supplementary information

Supplemental legends

Figure S1. 23 high-risk CLL pedigrees
