# Supplementary material for: Elevated IgM and abnormal free light chain ratio are increased in relatives from high-risk chronic lymphocytic leukemia pedigrees
Source: Blood Cancer J. 2019 Feb 26;9(3):25. doi: 10.1038/s41408-019-0186-8 (PMC6391432; doi:10.1038/s41408-019-0186-8)
Supplement: Supplementary file 1 — Supplemental legends [file 41408_2019_186_MOESM1_ESM.docx]

**Supplemental Figure Legend**

**Figure S1: 23 high-risk CLL pedigrees.**

High-risk CLL pedigrees contain a statistical excess of individuals diagnosed with CLL or SLL compared to that expected (p<0.05). Pedigree structures are drawn to include all known CLL cases from the UPDB that defined their high-risk status, all siblings of CLLs, all sampled relatives in this study and their siblings, and all connecting relatives to the common ancestral founder/s. For pedigree members, all primary cancers confirmed by the UCR are indicated. Individuals without cancer in sibships are condensed to a single diamond symbol containing the number of individuals condensed. Otherwise circles indicate females and squares indicate males. The right side of a symbol is shaded blue for relatives whose serum was tested and found to be below the 95^th^ percentile for IgM(κ+λ) (<194 mg/dL) and within the normal range for free light chain (FLC) ratio (0.26-1.65). The right side is shaded red for relatives whose serum was identified as elevated for IgM or abnormal for FLC ratio. Upper left quandrant of symbols are shaded black for CLL or SLL (proband cancers for the pedigrees) or grey for screen-detected monoclonal B-cell lymphocytosis (MBL). Lower left quadrant of symbols are shaded black for other hematological malignancies or grey for solid tumor cancers. Cancer site abbreviations are shown below symbols, and are as follows: ALL acute lymphocytic leukemia; AML=acute myeloid leukemia; BRE=breast cancer; CLL=chronic lymphocytic leukemia; CML=chronic myeloid leukemia; CNS=central nervous system cancers; CRC=colorectal cancers; DLB=diffuse large B-cell lymphoma; ERC=endocrine-related cancers; FL=follicular lymphoma; GI=gastrointestinal cancers; GYN=gynaecological cancers; HCL=hairy cell leukemia; HNC=head and neck cancers; LIV=liver cancer; LK=leukemia, not otherwise specified; LUN=lung cancer; MCL=Mantel cell lymphoma; MEL=melanoma; MM=multiple myeloma; MZL=maginal zone lymphoma; NHL=non-Hodgkin lymphoma, not otherwise specified; NMSC=non-melanoma skin cancer; PAN=pancreas; PCN=plasma cell neoplasm, not otherwise specified; PRO=prostate cancer; SAR=sarcomas; SLL=small lymphocytic lymphoma; URN=urinary system cancers; UNK=cancer of unknown type.
