# Supplementary material for: Elevated IgM and abnormal free light chain ratio are increased in relatives from high-risk chronic lymphocytic leukemia pedigrees
Source: Blood Cancer J. 2019 Feb 26;9(3):25. doi: 10.1038/s41408-019-0186-8 (PMC6391432; doi:10.1038/s41408-019-0186-8)

Figure S1: 23 high-risk CLL pedigrees

Disease abbreviations:

**ALL**	acute lymphocytic leukemia
**AML**	acute myeloid leukemia
**BRE**	breast cancer
**CLL**	chronic lymphocytic leukemia
**CML**	chronic myeloid leukemia
**CNS**	central nervous system cancers
**CRC**	colorectal cancer
**DLB**	diffuse large B-cell lymphoma
**ERC**	endocrine-related cancers
**FL**	follicular lymphoma
**GI**	gastrointestinal cancers
**GYN**	gynaecological cancers
**HCL**	hairy cell leukemia
**HNC**	head and neck cancer
**LIV**	liver cancer
**LK**	leukemia, not otherwise specified
**LUN**	lung cancer
**MBL**	monoclonal B-cell lymphocytosis
**MCL**	mantle cell lymphoma
**MEL**	melanoma
**MM**	multiple myeloma
**MZL**	marginal zone lymphoma
**NHL**	non-Hodgkin lymphoma, not otherwise specified
**NMSC**	non-melanoma skin cancer
**PAN**	pancreatic cancer
**PCN**	plasma cell neoplasm, not otherwise specified
**PRO**	prostate cancer
**SAR**	sarcoma
**SLL**	small lymphocytic lymphoma
**URN**	urinary system cancers
**UNK**	cancer of unknown type

Pedigree symbols:

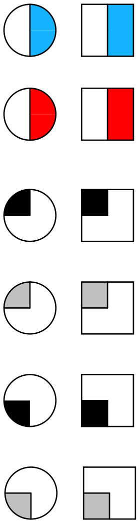

Sampled relative

Abnormal FLC ratio (<0.26 or >1.65) or elevated IgM (>194 mg/dL)

CLL/SLL

MBL

Other heme malignancy

Solid cancer

6204

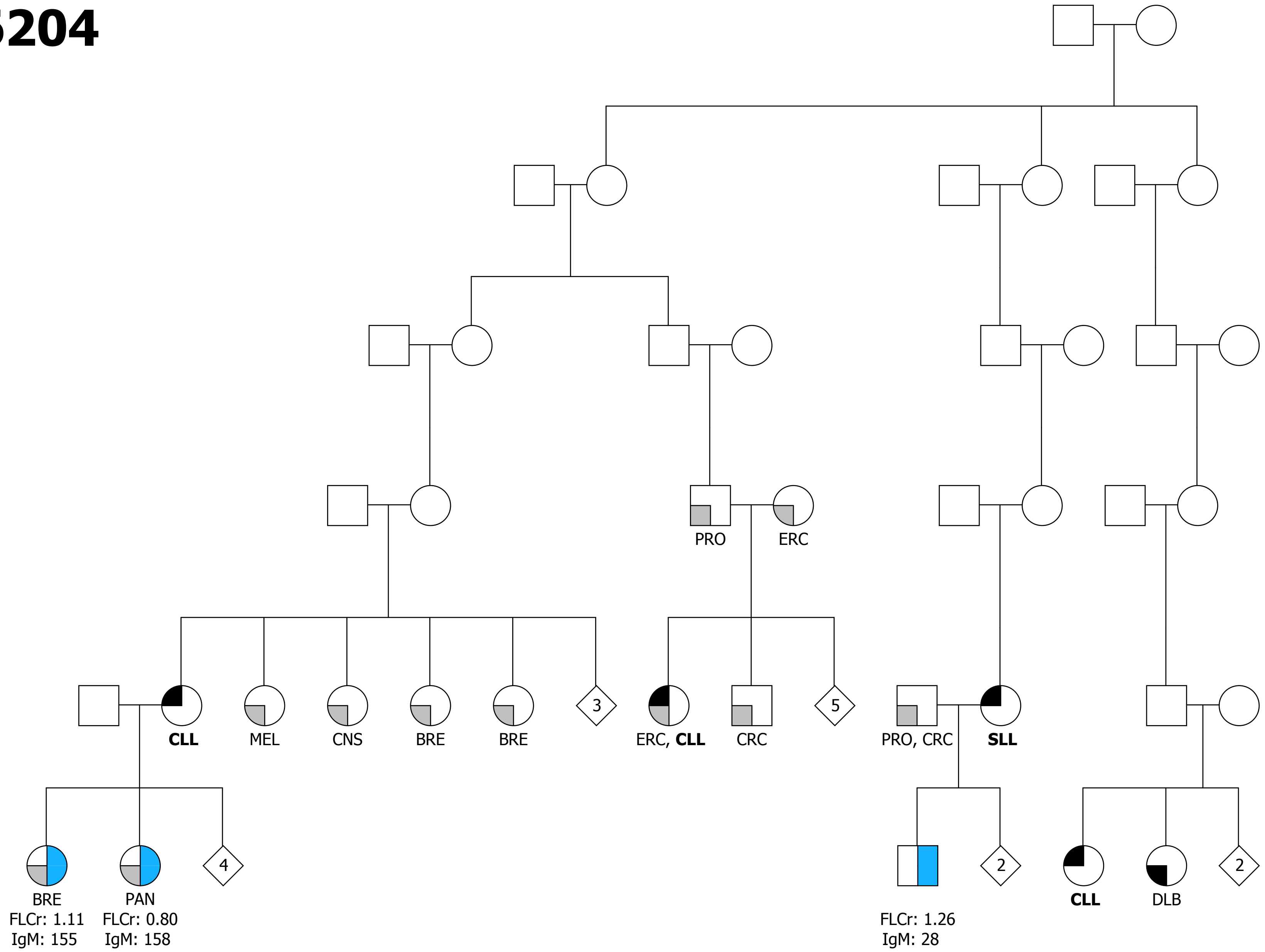
PRO
ERC
CLL
MEL
CNS
BRE
BRE
3
ERC, CLL
CRC
5
PRO, CRC
SLL
BRE
FLCr: 1.11
IgM: 155
PAN
FLCr: 0.80
IgM: 158
4
FLCr: 1.26
IgM: 28
2
CLL
DLB
2

6205

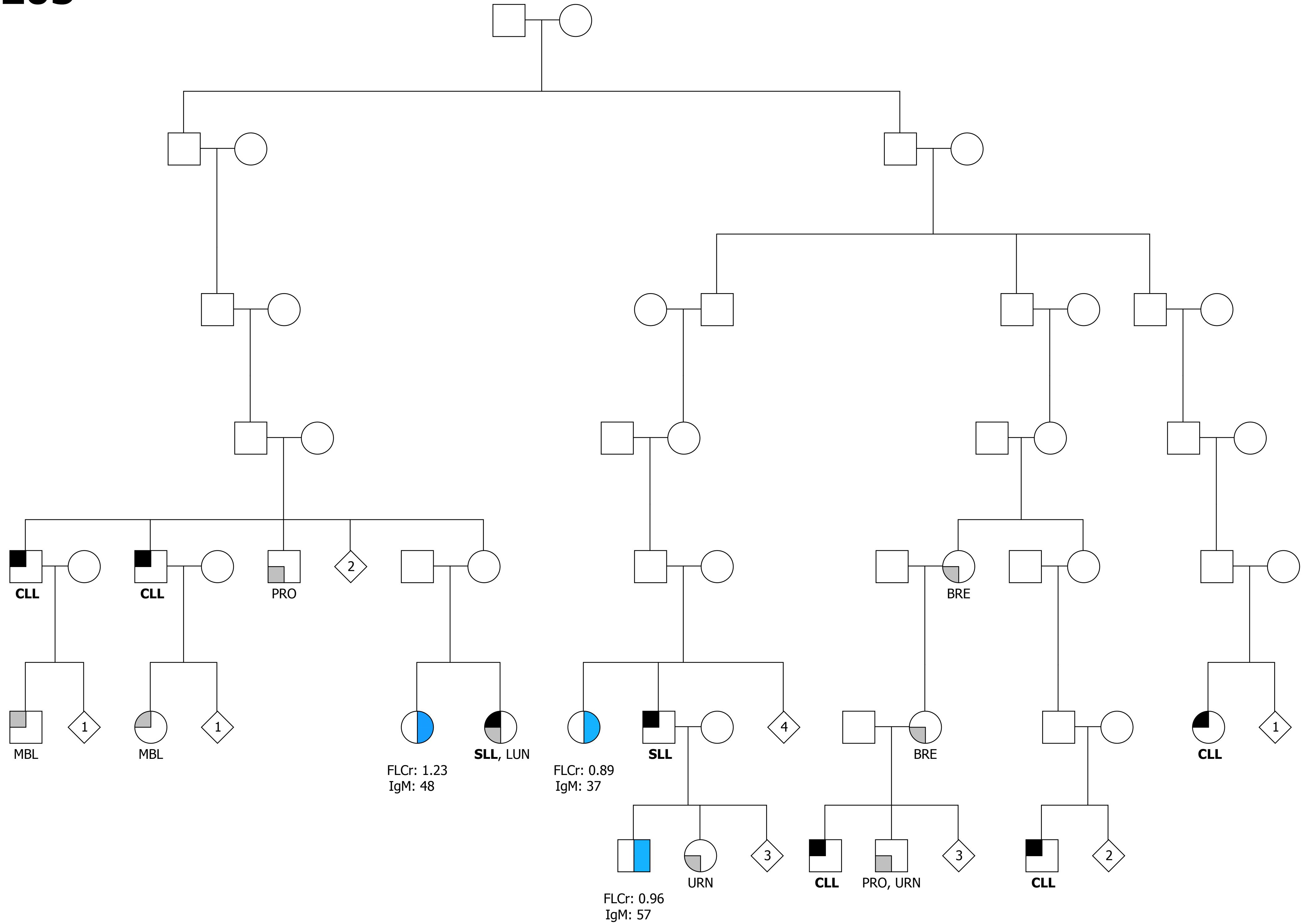
CLL
CLL
PRO
2
BRE
MBL
1
MBL
1
FLCr: 1.23
IgM: 48
SLL, LUN
FLCr: 0.89
IgM: 37
SLL
4
BRE
CLL
1
FLCr: 0.96
IgM: 57
URN
3
CLL
PRO, URN
3
CLL
2

6207

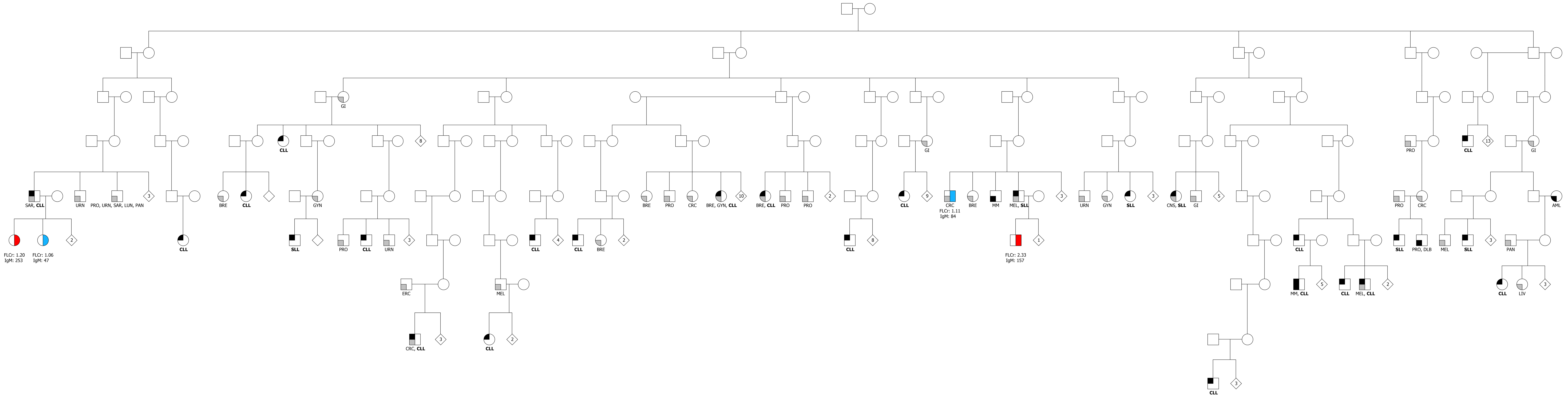
GI
8
CLL
PRO
CLL
13
GI
SAR, CLL
URN
PRO, URN, SAR, LUN, PAN
3
BRE
CLL
GYN
BRE
PRO
CRC
BRE, GYN, CLL
10
BRE, CLL
PRO
PRO
2
CLL
9
CRC
FLCr: 1.11
IgM: 84
BRE
MM
MEL, SLL
3
URN
GYN
SLL
3
CNS, SLL
GI
5
PRO
CRC
AML
FLCr: 1.20
IgM: 253
FLCr: 1.06
IgM: 47
2
CLL
SLL
PRO
CLL
URN
3
CLL
4
CLL
BRE
2
CLL
8
FLCr: 2.33
IgM: 157
1
CLL
SLL
PRO, DLB
MEL
SLL
3
PAN
ERC
MEL
MM, CLL
5
CLL
MEL, CLL
2
CLL
LIV
3
CRC, CLL
3
CLL
2
CLL
3

6208

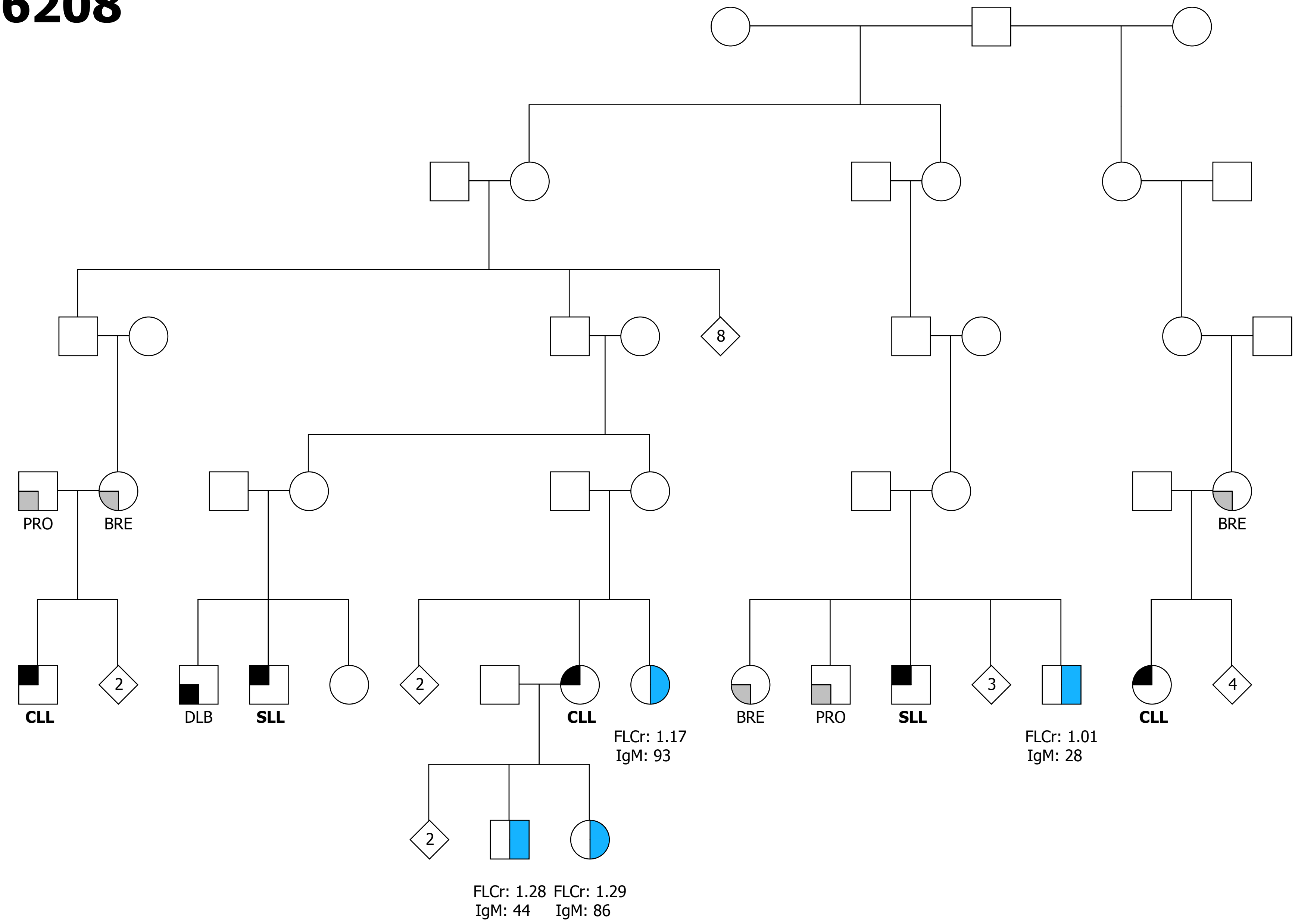
8
PRO
BRE
BRE
2
DLB
CLL
SLL
2
CLL
FLCr: 1.17
IgM: 93
BRE
PRO
SLL
3
FLCr: 1.01
IgM: 28
CLL
4
2
FLCr: 1.28
IgM: 44
FLCr: 1.29
IgM: 86

6210

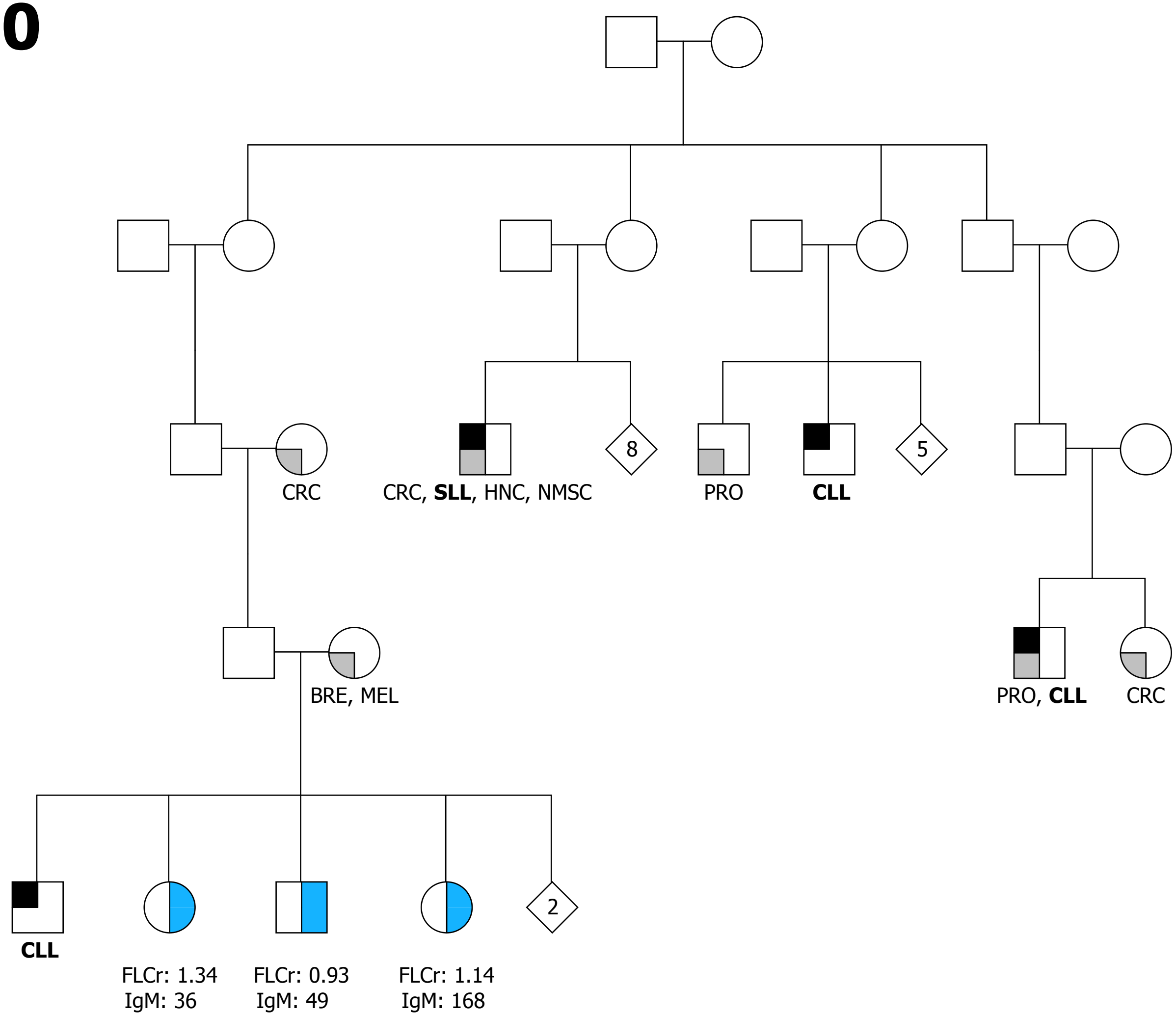

8
5
CRC
CRC, **SLL**, HNC, NMSC
PRO
CLL
BRE, MEL
PRO, **CLL**
CRC
2
CLL
FLCr: 1.34
IgM: 36
FLCr: 0.93
IgM: 49
FLCr: 1.14
IgM: 168

6211

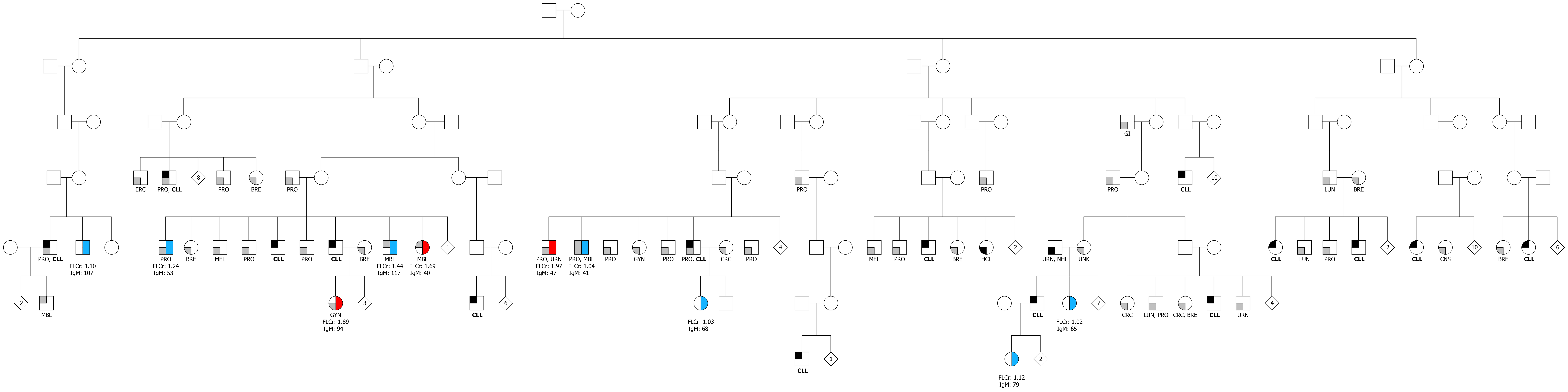

ERC
PRO, CLL
8
PRO
BRE
PRO
PRO
GI
PRO
PRO
PRO
CLL
10
LUN
BRE
PRO, CLL
FLCr: 1.10
IgM: 107
PRO
FLCr: 1.24
IgM: 53
BRE
MEL
PRO
CLL
PRO
CLL
BRE
MBL
FLCr: 1.44
IgM: 117
MBL
FLCr: 1.69
IgM: 40
1
PRO, URN
FLCr: 1.97
IgM: 47
PRO, MBL
FLCr: 1.04
IgM: 41
PRO
GYN
PRO
PRO, CLL
CRC
PRO
4
MEL
PRO
CLL
BRE
HCL
2
URN, NHL
UNK
CLL
LUN
PRO
CLL
2
CLL
CNS
10
BRE
CLL
6
2
MBL
GYN
FLCr: 1.89
IgM: 94
3
CLL
6
FLCr: 1.03
IgM: 68
CLL
FLCr: 1.02
IgM: 65
7
CRC
LUN, PRO
CRC, BRE
CLL
URN
4
CLL
1
FLCr: 1.12
IgM: 79
2

6213

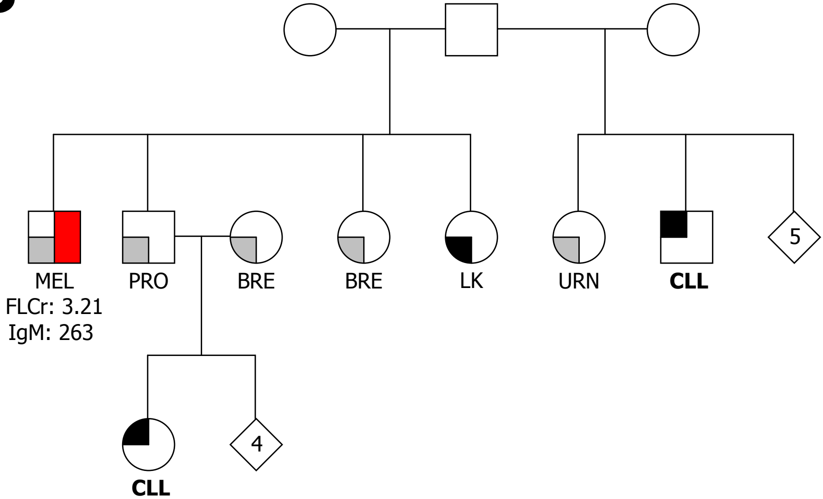

6215

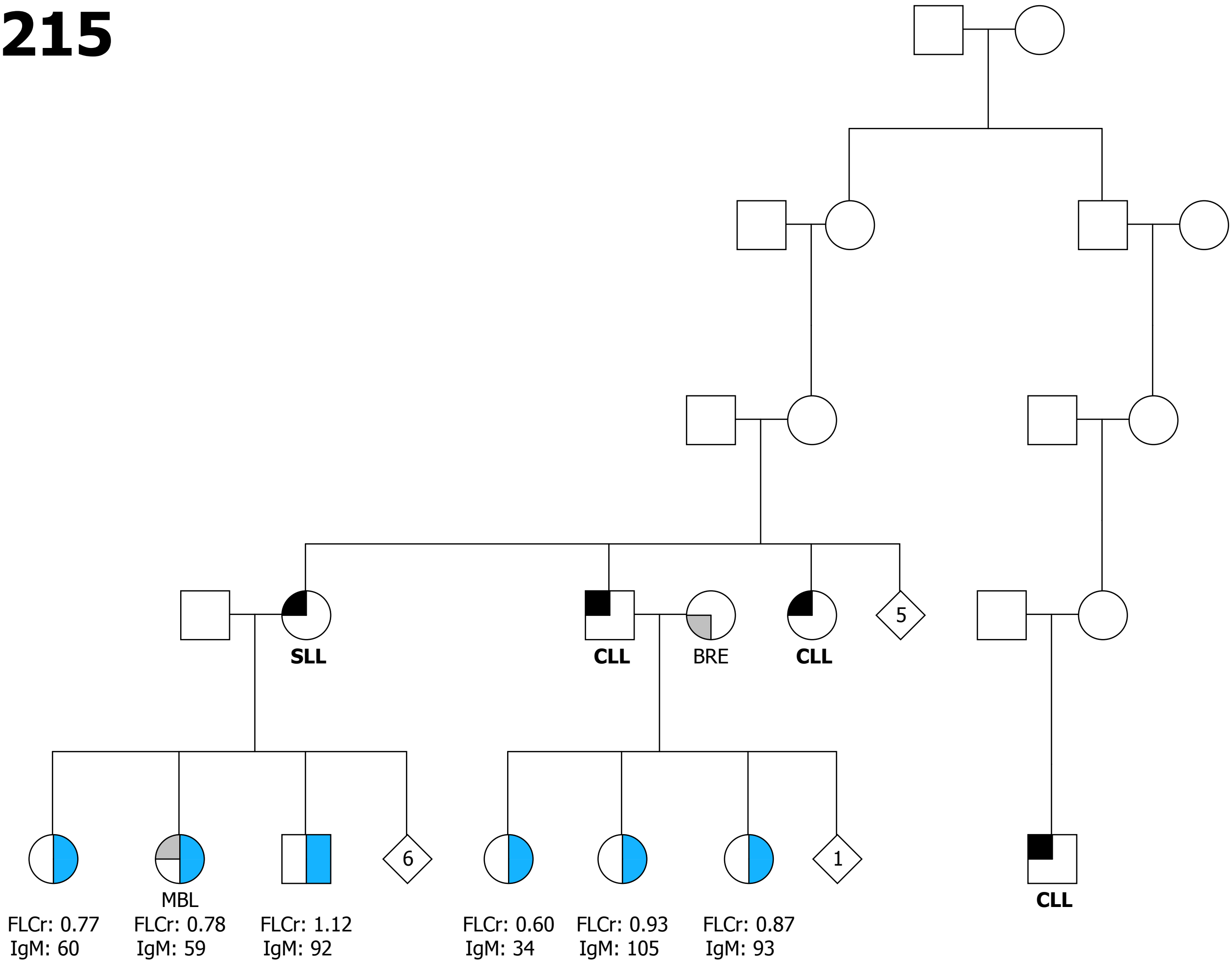

5
SLL
CLL
BRE
CLL
6
1
MBL
FLCr: 0.77
IgM: 60
FLCr: 0.78
IgM: 59
FLCr: 1.12
IgM: 92
FLCr: 0.60
IgM: 34
FLCr: 0.93
IgM: 105
FLCr: 0.87
IgM: 93
CLL

6217

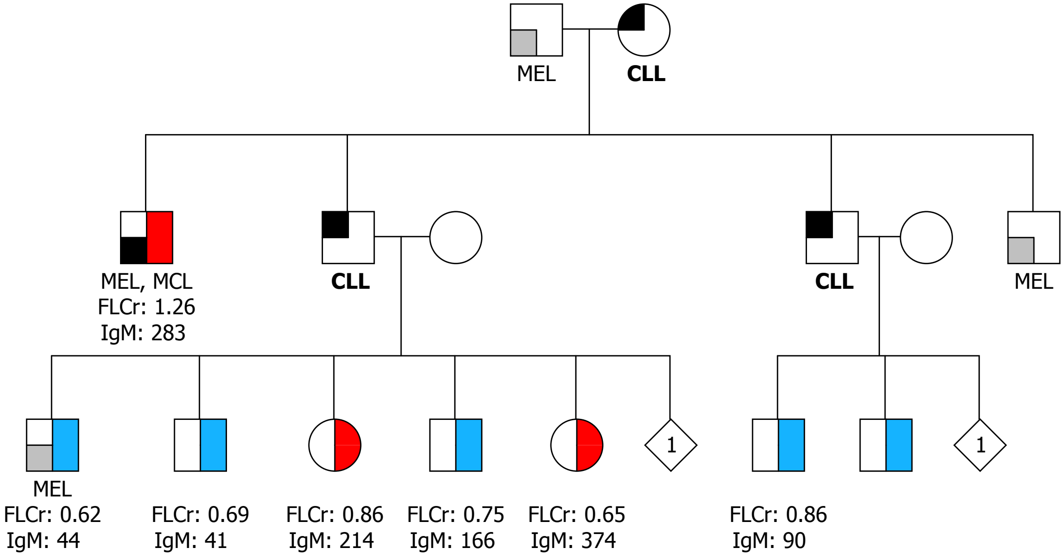
MEL
CLL
MEL, MCL
FLCr: 1.26
IgM: 283
CLL
CLL
MEL
1
1
MEL
FLCr: 0.62
IgM: 44
FLCr: 0.69
IgM: 41
FLCr: 0.86
IgM: 214
FLCr: 0.75
IgM: 166
FLCr: 0.65
IgM: 374
FLCr: 0.86
IgM: 90

6218

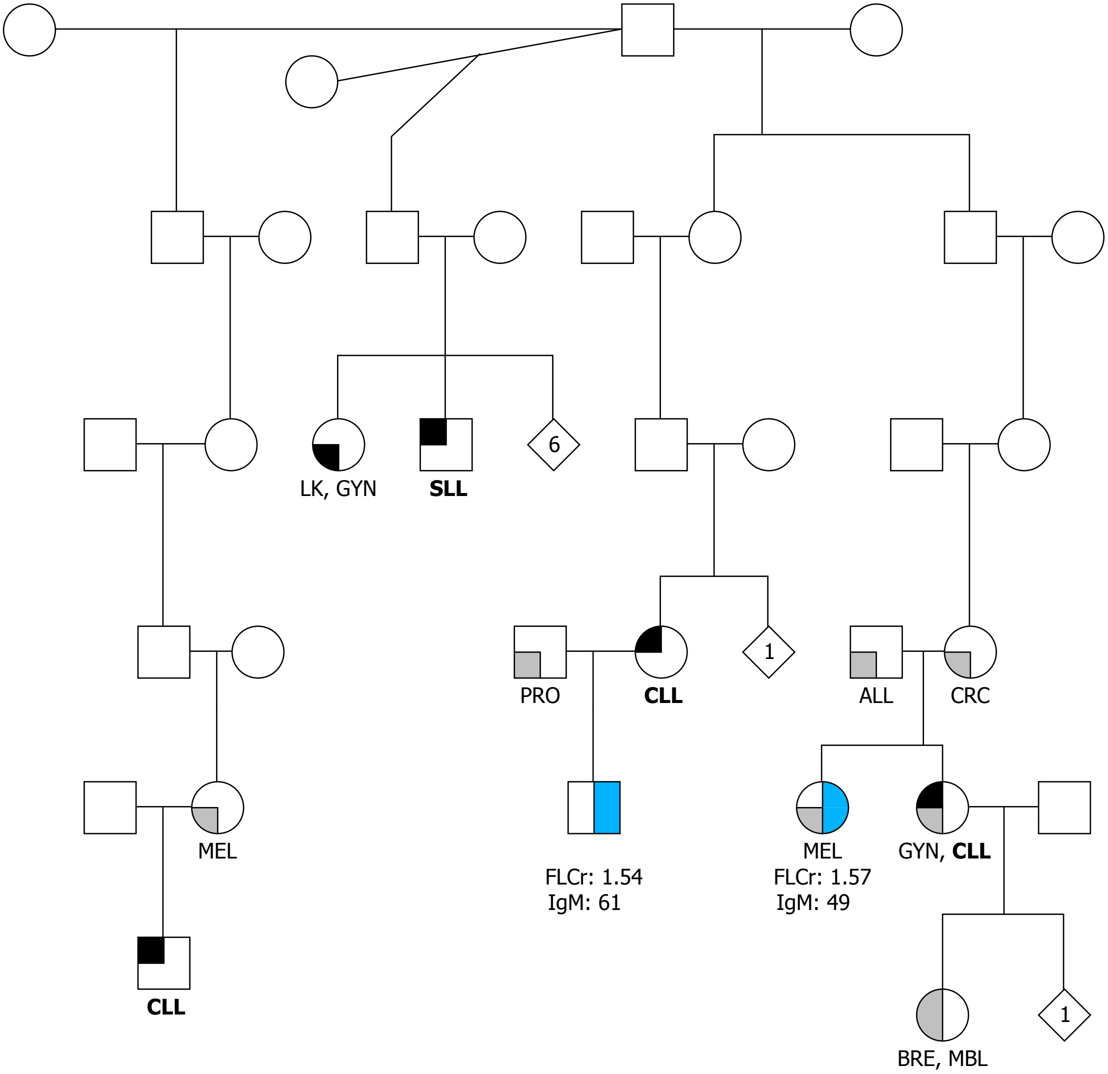

6
LK, GYN
SLL
1
PRO
CLL
ALL
CRC
MEL
FLCr: 1.54
IgM: 61
MEL
FLCr: 1.57
IgM: 49
GYN, CLL
CLL
1
BRE, MBL

6222

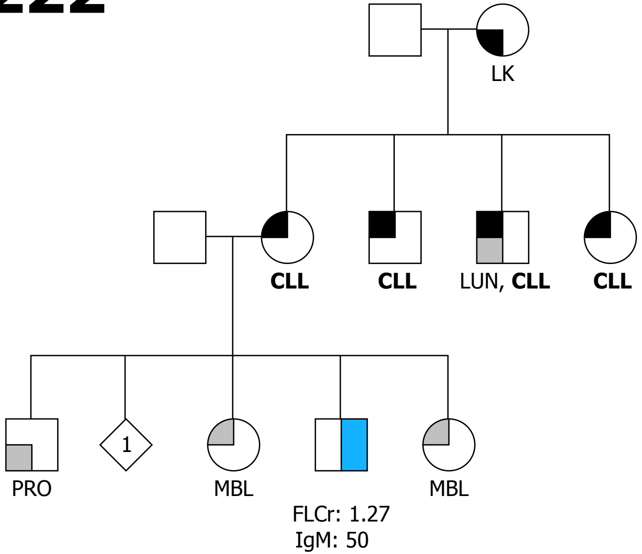

6223

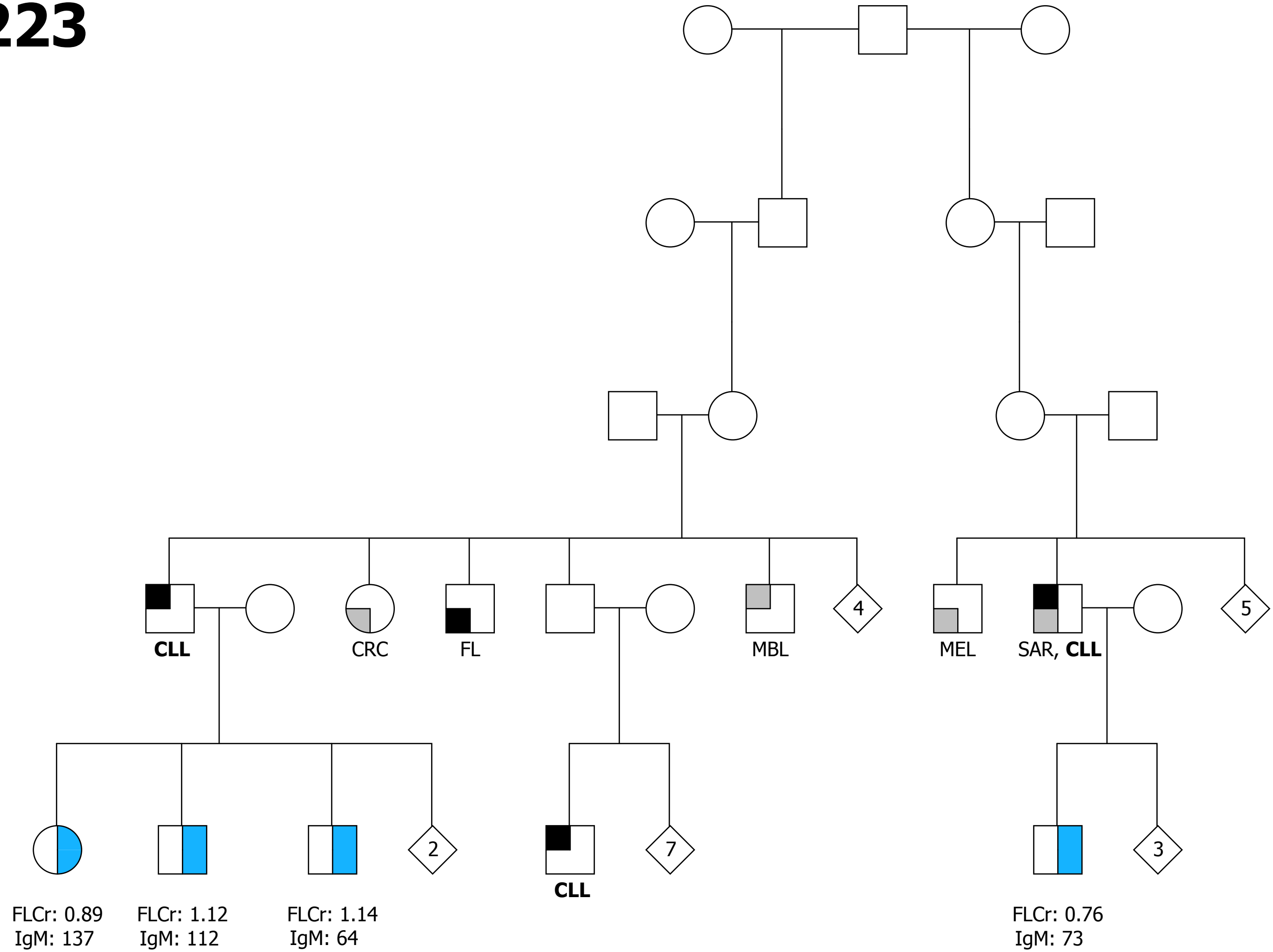

4
CLL
CRC
FL
MBL
MEL
SAR, CLL
5
2
7
3
CLL
FLCr: 0.89
IgM: 137
FLCr: 1.12
IgM: 112
FLCr: 1.14
IgM: 64
FLCr: 0.76
IgM: 73

6224

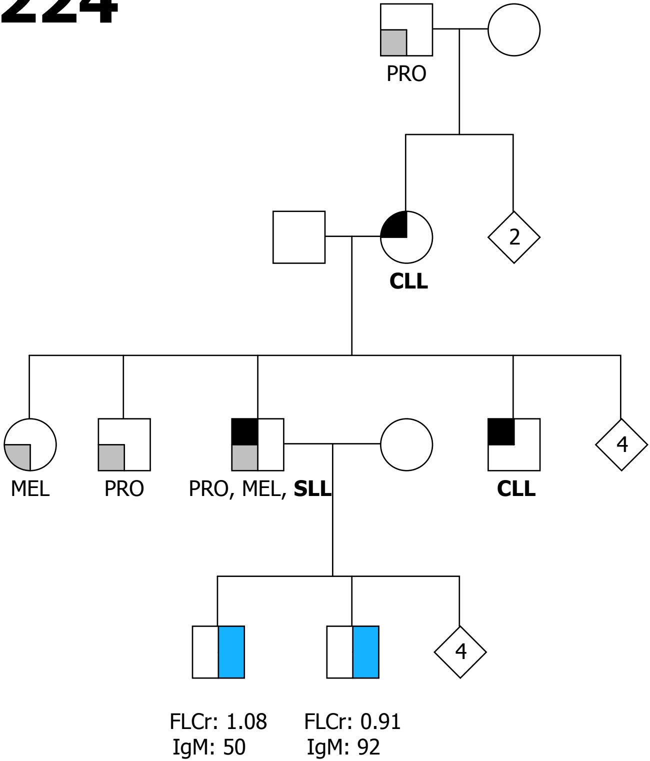
PRO
CLL
2
MEL
PRO
PRO, MEL, SLL
CLL
4
4
FLCr: 1.08
IgM: 50
FLCr: 0.91
IgM: 92

6225

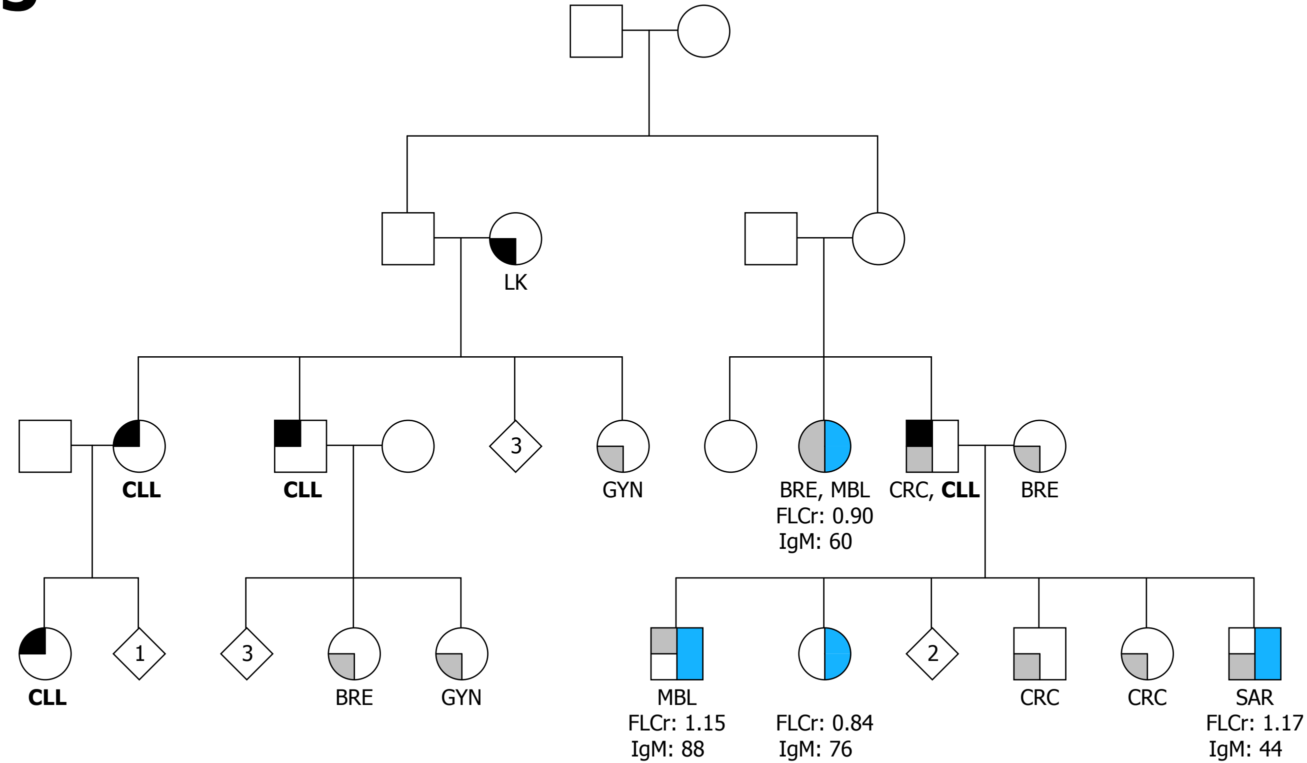

LK
3
CLL
CLL
GYN
BRE, MBL
FLCr: 0.90
IgM: 60
CRC, CLL
BRE
1
3
2
CLL
BRE
GYN
MBL
FLCr: 1.15
IgM: 88
FLCr: 0.84
IgM: 76
CRC
CRC
SAR
FLCr: 1.17
IgM: 44

6226

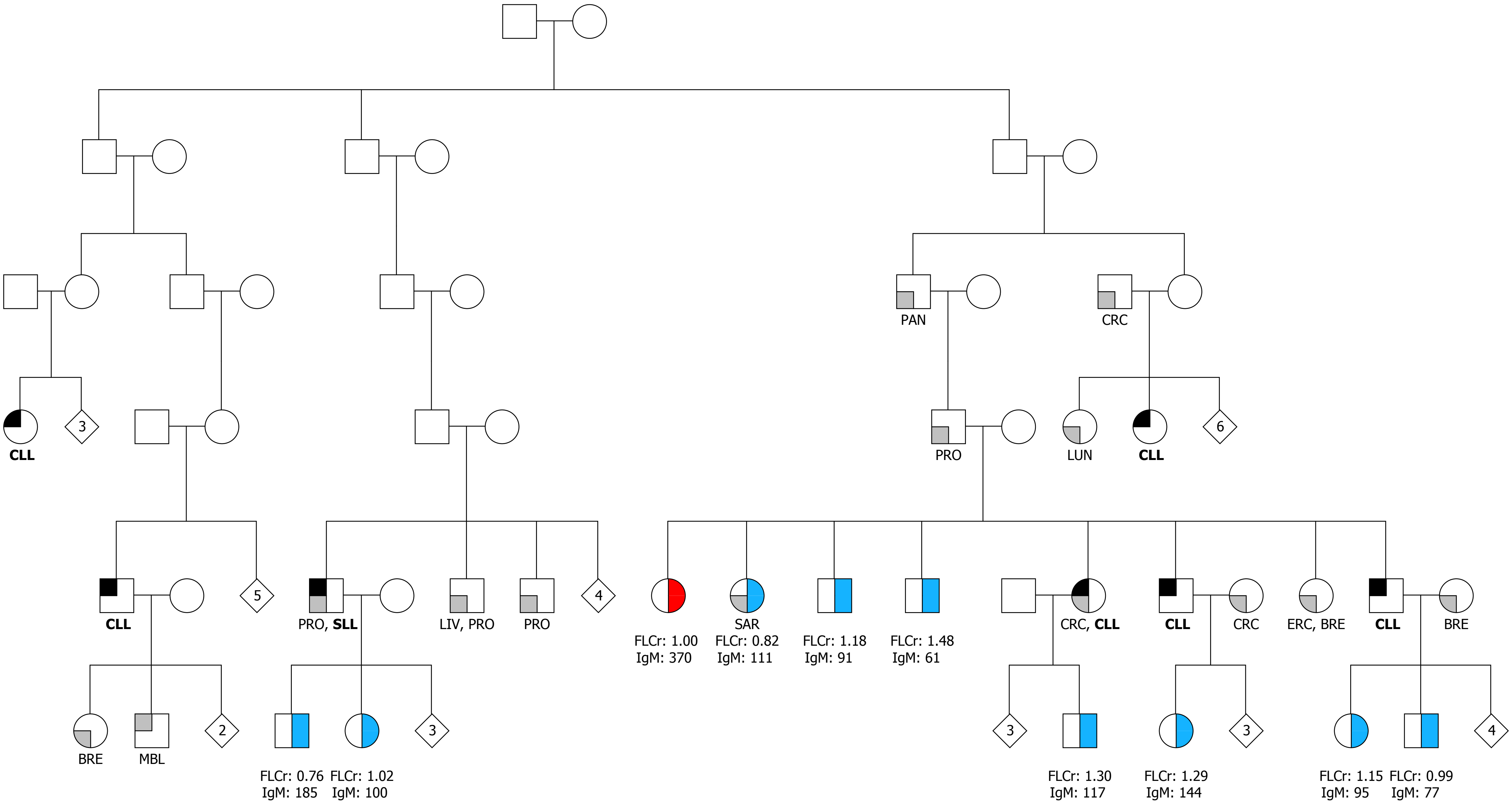
CLL
3
PAN
CRC
PRO
LUN
CLL
6
CLL
5
PRO, SLL
LIV, PRO
PRO
4
FLCr: 1.00
IgM: 370
SAR
FLCr: 0.82
IgM: 111
FLCr: 1.18
IgM: 91
FLCr: 1.48
IgM: 61
CRC, CLL
CLL
CRC
ERC, BRE
CLL
BRE
BRE
MBL
2
3
FLCr: 0.76
IgM: 185
FLCr: 1.02
IgM: 100
3
FLCr: 1.30
IgM: 117
FLCr: 1.29
IgM: 144
3
FLCr: 1.15
IgM: 95
FLCr: 0.99
IgM: 77
4

6228

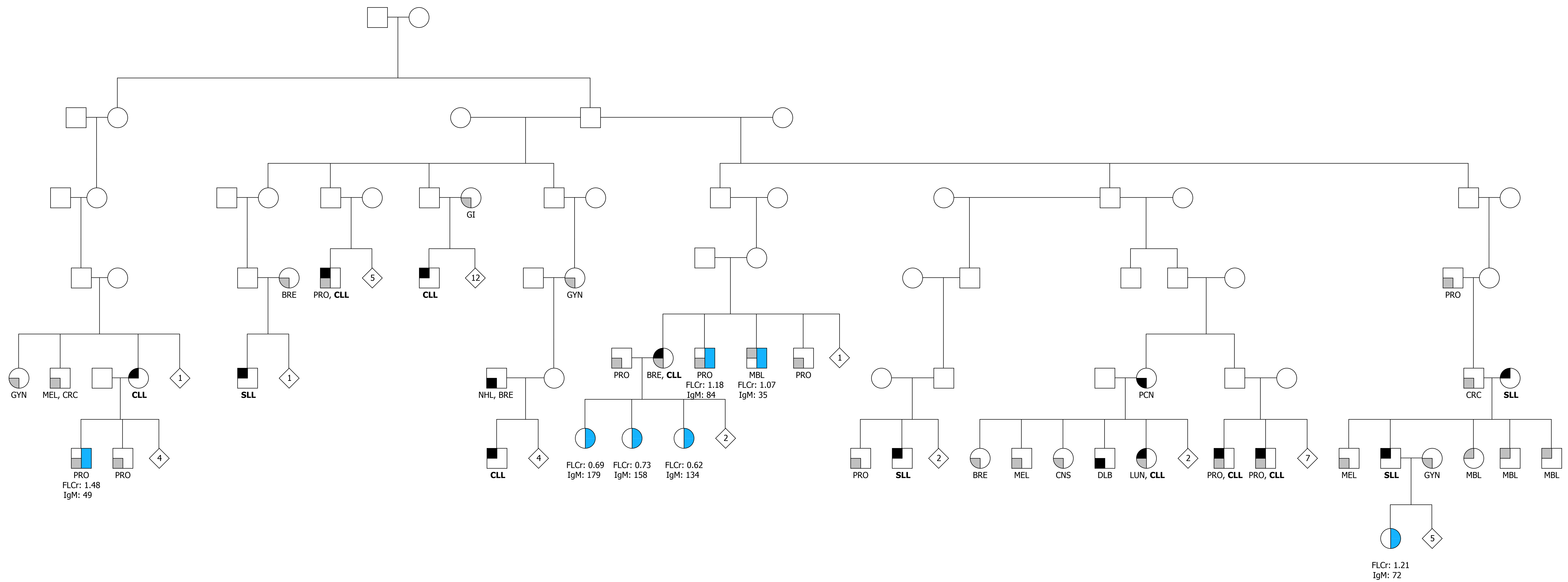

GI
BRE
PRO, CLL
CLL
GYN
PRO
GYN
MEL, CRC
CLL
SLL
NHL, BRE
PRO
BRE, CLL
PRO
FLCr: 1.18
IgM: 84
MBL
FLCr: 1.07
IgM: 35
PRO
PCN
CRC
SLL
PRO
FLCr: 1.48
IgM: 49
PRO
CLL
FLCr: 0.69
IgM: 179
FLCr: 0.73
IgM: 158
FLCr: 0.62
IgM: 134
PRO
SLL
BRE
MEL
CNS
DLB
LUN, CLL
PRO, CLL
PRO, CLL
MEL
SLL
GYN
MBL
MBL
MBL
FLCr: 1.21
IgM: 72

6229

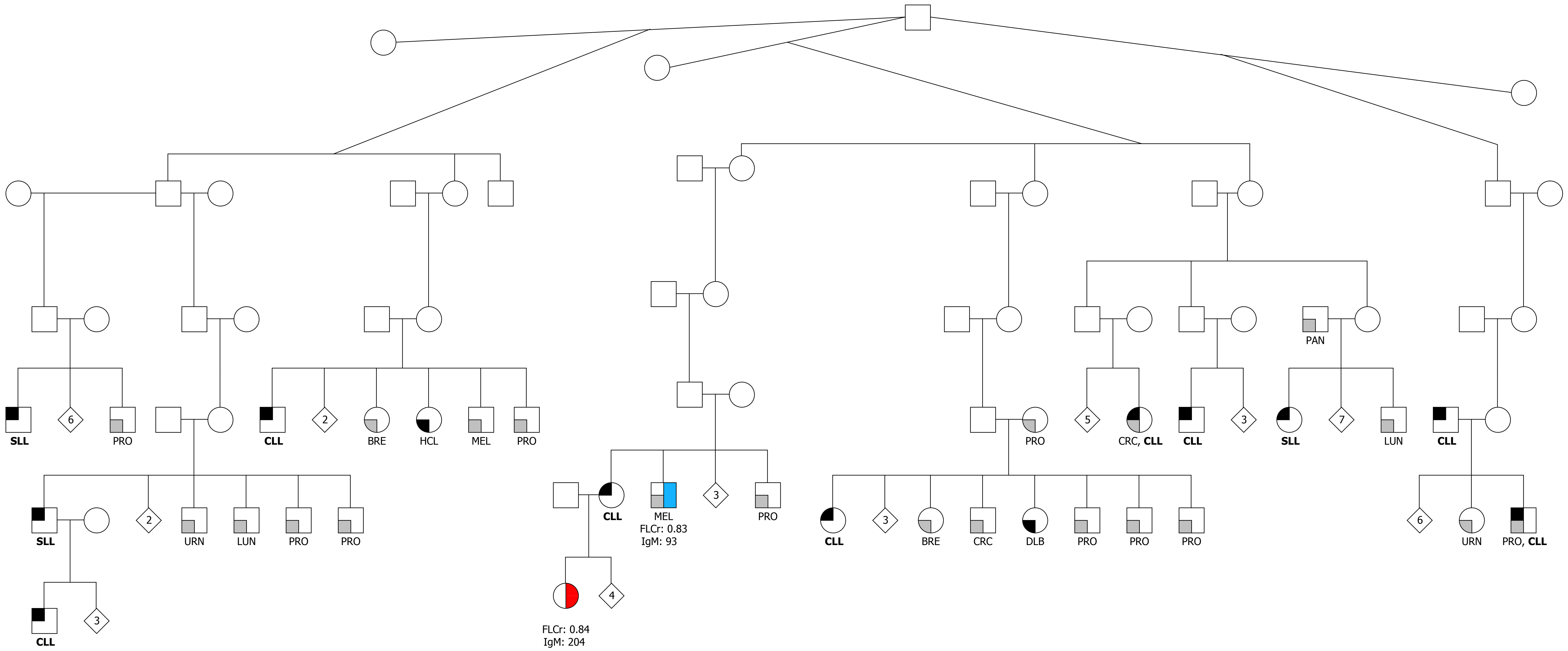
SLL
6
PRO
CLL
2
BRE
HCL
MEL
PRO
SLL
2
URN
LUN
PRO
PRO
CLL
3
CLL
MEL
FLCr: 0.83
IgM: 93
3
PRO
FLCr: 0.84
IgM: 204
4
PAN
PRO
5
CRC, CLL
CLL
3
SLL
7
LUN
CLL
CLL
3
BRE
CRC
DLB
PRO
PRO
PRO
6
URN
PRO, CLL

6232

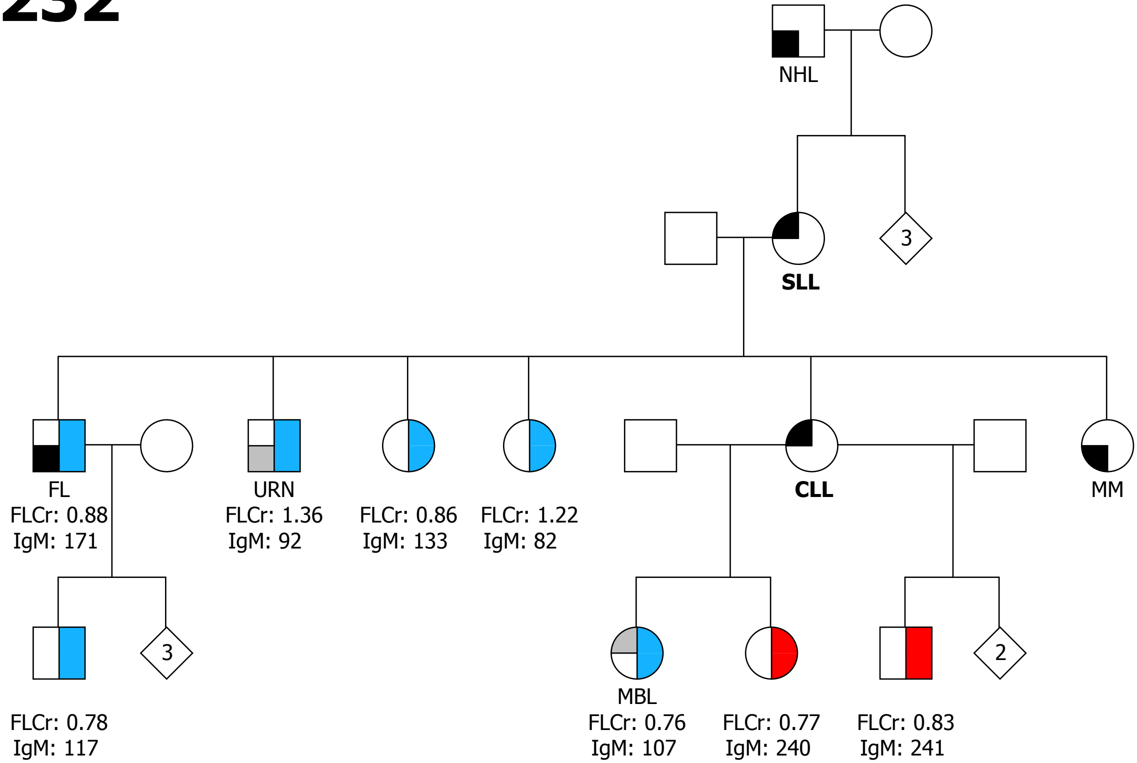
NHL
3
SLL
FL
FLCr: 0.88
IgM: 171
URN
FLCr: 1.36
IgM: 92
FLCr: 0.86
IgM: 133
FLCr: 1.22
IgM: 82
CLL
MM
3
FLCr: 0.78
IgM: 117
MBL
FLCr: 0.76
IgM: 107
FLCr: 0.77
IgM: 240
FLCr: 0.83
IgM: 241
2

6233

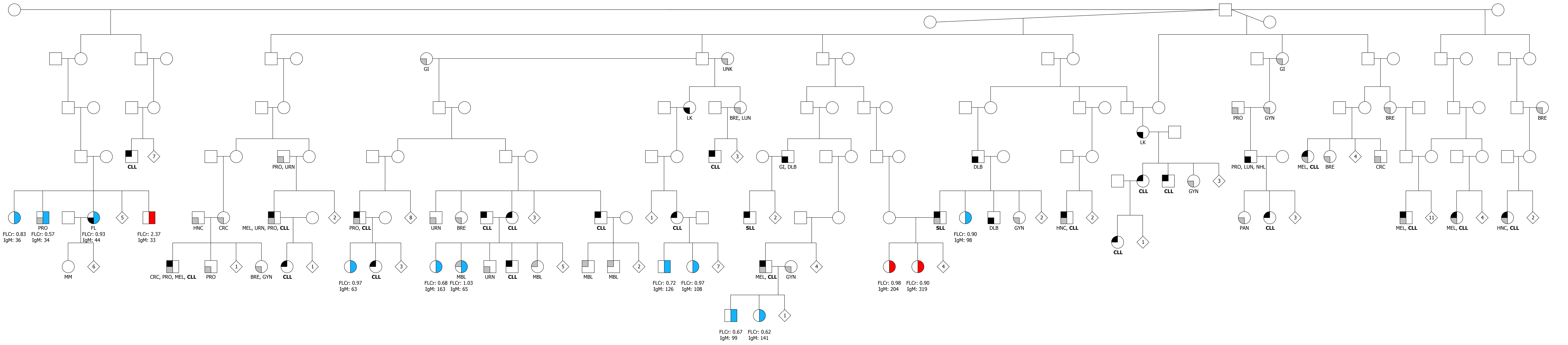
GI
UNK
LK
BRE, LUN
PRO
GYN
GI
BRE
BRE
CLL
PRO, URN
CLL
GI, DLB
DLB
LK
PRO, LUN, NHL
MEL, CLL
BRE
CRC
CLL
CLL
GYN
FLCr: 0.83
IgM: 36
PRO
FLCr: 0.57
IgM: 34
FL
FLCr: 0.93
IgM: 44
FLCr: 2.37
IgM: 33
HNC
CRC
MEL, URN, PRO, CLL
PRO, CLL
URN
BRE
CLL
CLL
CLL
CLL
SLL
SLL
FLCr: 0.90
IgM: 98
DLB
GYN
HNC, CLL
PAN
CLL
MEL, CLL
MEL, CLL
HNC, CLL
MM
CRC, PRO, MEL, CLL
PRO
BRE, GYN
CLL
FLCr: 0.97
IgM: 63
CLL
FLCr: 0.68
IgM: 163
MBL
FLCr: 1.03
IgM: 65
URN
CLL
MBL
MBL
MBL
FLCr: 0.72
IgM: 126
FLCr: 0.97
IgM: 108
MEL, CLL
GYN
FLCr: 0.98
IgM: 204
FLCr: 0.90
IgM: 319
CLL
FLCr: 0.67
IgM: 99
FLCr: 0.62
IgM: 141

6234

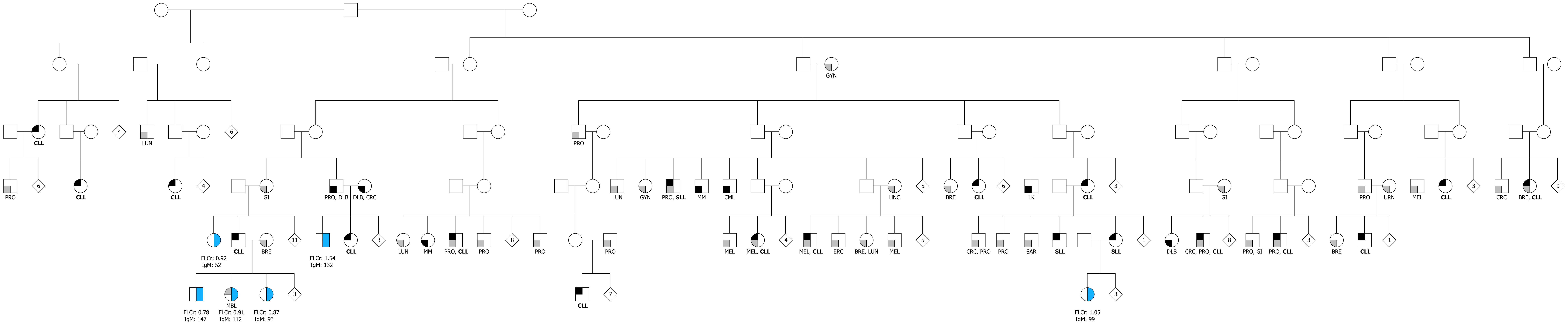
CLL
4
LUN
6
PRO
6
CLL
CLL
4
GYN
PRO
GI
PRO, DLB
DLB, CRC
FLCr: 0.92
IgM: 52
CLL
BRE
11
FLCr: 1.54
IgM: 132
CLL
3
FLCr: 0.78
IgM: 147
MBL
FLCr: 0.91
IgM: 112
FLCr: 0.87
IgM: 93
3
LUN
MM
PRO, CLL
PRO
8
PRO
PRO
CLL
7
LUN
GYN
PRO, SLL
MM
CML
HNC
5
MEL
MEL, CLL
4
MEL, CLL
ERC
BRE, LUN
MEL
5
BRE
CLL
6
LK
CLL
3
CRC, PRO
PRO
SAR
SLL
SLL
1
FLCr: 1.05
IgM: 99
3
GI
DLB
CRC, PRO, CLL
8
PRO, GI
PRO, CLL
3
PRO
URN
BRE
CLL
1
MEL
CLL
3
CRC
BRE, CLL
9

6235

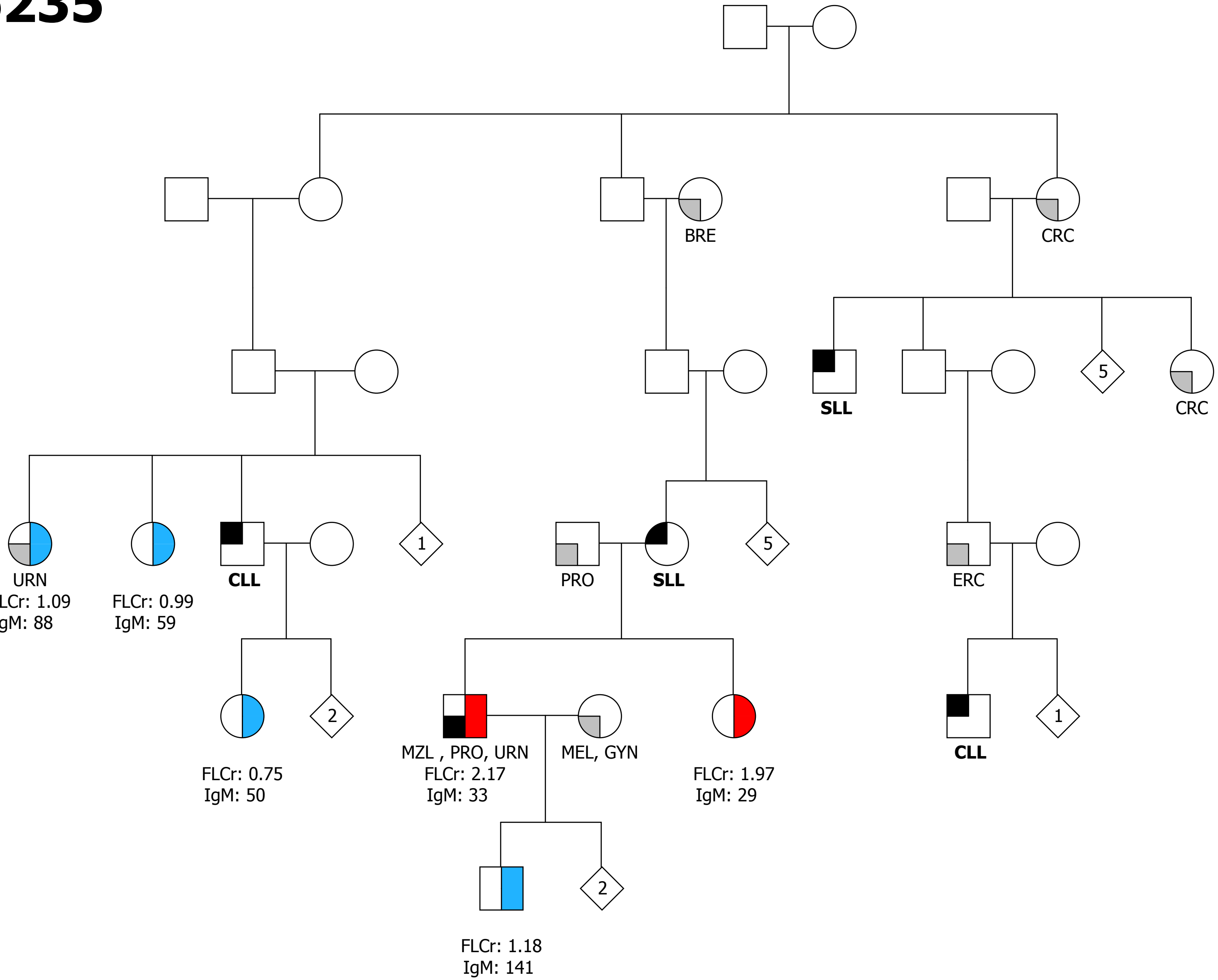
BRE
CRC
SLL
5
CRC
URN
FLCr: 1.09
IgM: 88
FLCr: 0.99
IgM: 59
CLL
1
PRO
SLL
5
ERC
2
MZL , PRO, URN
FLCr: 2.17
IgM: 33
MEL, GYN
FLCr: 1.97
IgM: 29
CLL
1
FLCr: 0.75
IgM: 50
2
FLCr: 1.18
IgM: 141

6236

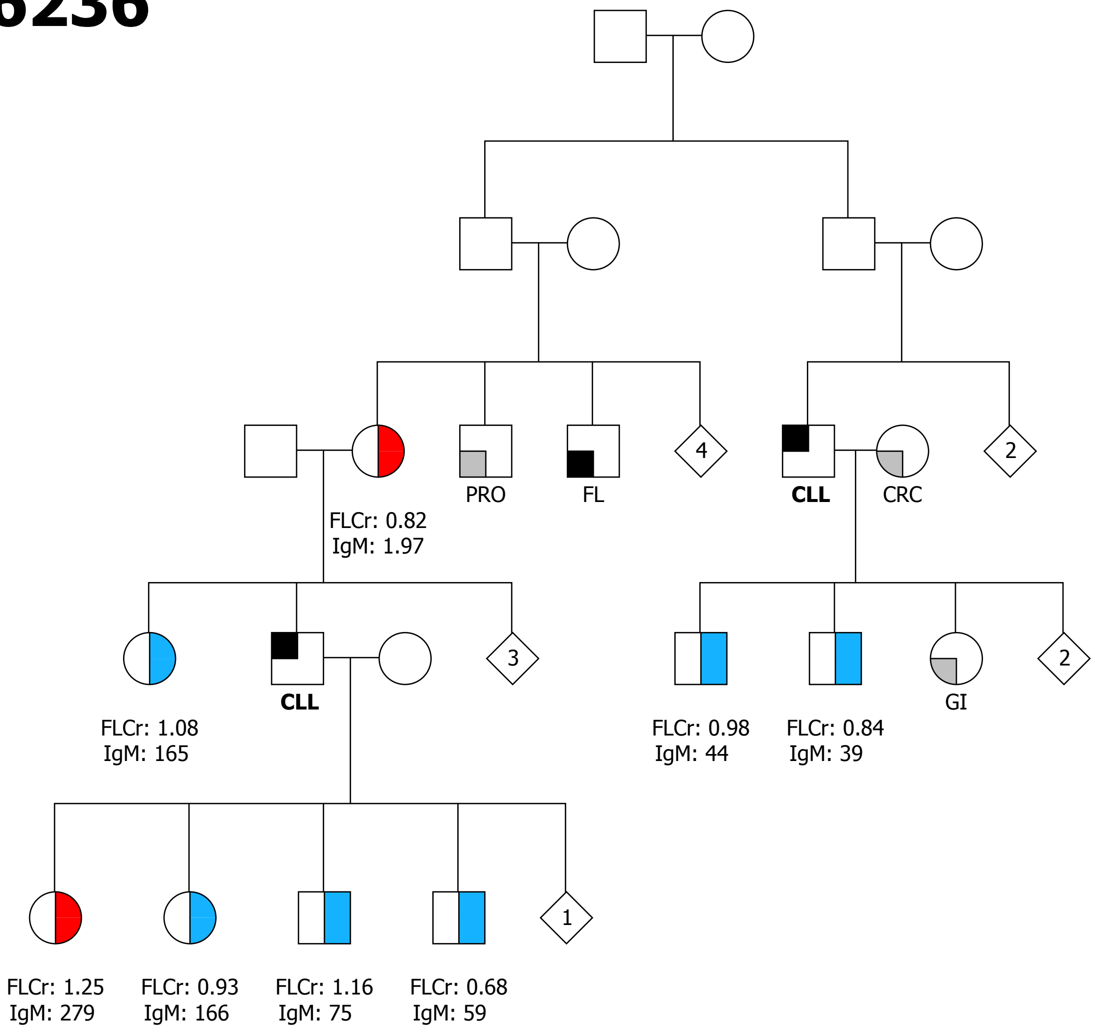

4
PRO
FL
CLL
CRC
2
FLCr: 0.82
IgM: 1.97
3
CLL
FLCr: 1.08
IgM: 165
GI
2
FLCr: 0.98
IgM: 44
FLCr: 0.84
IgM: 39
1
FLCr: 1.25
IgM: 279
FLCr: 0.93
IgM: 166
FLCr: 1.16
IgM: 75
FLCr: 0.68
IgM: 59

6239

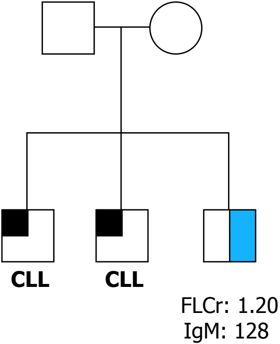

Supplement: Supplementary file 2 — Figure S1. 23 high-risk CLL pedigrees [file 41408_2019_186_MOESM2_ESM.pdf]
